# CmMLO17 and its partner CmKIC potentially support *Alternaria alternata* growth in *Chrysanthemum morifolium*

**DOI:** 10.1038/s41438-021-00534-x

**Published:** 2021-05-01

**Authors:** Jingjing Xin, Ye Liu, Huiyun Li, Sumei Chen, Jiafu Jiang, Aiping Song, Weimin Fang, Fadi Chen

**Affiliations:** State Key Laboratory of Crop Genetics and Germplasm Enhancement, Key Laboratory of Landscaping, Ministry of Agriculture and Rural Affairs, College of Horticulture, Nanjing Agricultural University, Nanjing, 210095 China

**Keywords:** RNAi, Fungi

## Abstract

The *Mildew Resistance Locus O* (*MLO*) gene family has been investigated in many species. However, there are few studies on chrysanthemum *MLO* genes. We report in this study that *CmMLO17* in *Chrysanthemum morifolium* was upregulated after *Alternaria alternata* infection. Silencing of *CmMLO17* by artificial microRNA resulted in reduced susceptibility of chrysanthemum to *A. alternata* infection. Genes in the abscisic acid (ABA) and Ca^2+^ signaling pathways were upregulated in the *CmMLO17*-silenced line R20 compared to the wild-type plants. We speculated that *CmMLO17*-silenced plants had a faster and stronger defense response that was mediated by the ABA and Ca^2+^ signaling pathways, resulting in reduced susceptibility of chrysanthemum to *A. alternata* infection. In addition, a candidate gene, *CmKIC*, that may interact with CmMLO17 was discovered by the yeast two-hybrid assay. The interaction between CmMLO17 and CmKIC was confirmed using the yeast two-hybrid assay and bimolecular fluorescence complementation (BiFC) analysis. CmMLO17 and CmKIC were both located on the plasma membrane, and CmKIC was also located on the nucleus. *CmKIC* overexpression increased the susceptibility of chrysanthemum to *A. alternata*, whereas *CmKIC* silencing resulted in reduced susceptibility. Therefore, CmMLO17 and CmKIC may work together in *C. morifolium* to support the growth of *A. alternata*. The results of this study will provide insight into the potential function of MLO and improve the understanding of plant defense responses to necrotrophic pathogens.

## Introduction

Chrysanthemum (*Chrysanthemum morifolium*) is one of the most common cut flowers in the world and possesses ornamental and economic value. Leaf black spot disease, caused by the necrotrophic fungus *Alternaria alternata*, is one of the most serious diseases during chrysanthemum production. After *A. alternata* infection, the chrysanthemum leaves exhibit round black spots, which gradually expand to round, nearly round, or irregular spots, and dark mildew spots are formed under humid conditions. Recently, the incidence of black spot disease has increased, which has severely affected the output and ornamental quality of chrysanthemum and caused great losses in flower production. At present, black spot disease is mainly controlled by the spraying of fungicides; however, this often causes environmental pollution and increases the resistance of fungal pathogens to pesticides. Therefore, breeding resistant varieties are the most economical, safe, and effective way to control black spot disease in chrysanthemum.

One method of breeding disease-resistant chrysanthemum is based on the introduction of the R gene, which encodes a protein that recognizes the effectors of pathogens and triggers a series of defense responses^1^. Activation of the R gene produces reactive oxygen species (ROS) and a hypersensitive response at the sites of pathogenic infection. This is a programmed cell death response that can prevent further invasion by pathogens^2–5^. However, new mutants of pathogens can overcome R gene-mediated resistance, and the persistence of R genes is limited^6^. An alternative method is to modify susceptibility genes (S genes), the absence or silencing of which reduces plant susceptibility to pathogens^7^.

The *Mildew Resistance Locus O* (*MLO*) is a typical class of S genes that contributes to the infection and growth of powdery mildew (PM). First discovered in barley, loss-of-function mutants of the *MLO* gene have broad-spectrum resistance to almost all known pathovars of the PM pathogen *Blumeria graminis* f. sp. *hordei*^8^. Many studies have revealed that *MLO* genes are highly conserved throughout the plant kingdom, and their loss-of-function mutants make plants, such as *Arabidopsis thaliana*^9^, tomato^10^, pea^11^, pepper^12^, wheat^13^, apple^14^, and grapevine^15^, resistant to PM. MLO proteins are categorized into seven phylogenetic branches^16,17^, of which only two clades include the S genes, and clades IV and V contain all S genes of monocots and dicots, respectively^9,10,18–21^. Transcription levels of S genes are upregulated during the early stages of pathogen invasion; therefore, not all members belonging to these two clades are S genes. Based on this characteristic, candidate genes can be identified, as has been reported in barley^22^, tomato^10^, pepper^12^, grape^15,20,21^, rose^23^, and apple^14^.

Topological analysis has shown that MLO is a plasma membrane protein with seven transmembrane domains; this protein is concentrated at the plasma membrane and has an extracellular amino terminus and an intracellular carboxy terminus^24^. The cytoplasmic C-terminus of MLO proteins harbors a calmodulin-binding domain (CaMBD), which is highly conserved across the protein family^25,26^. To be fully active, barley MLO requires the binding of CaM and CaMBD under Ca^2+^-dependent conditions^26^.

Previous studies have shown that a possible function of MLO proteins is to negatively regulate vesicle-related and actin-dependent defense responses at sites where pathogens attempt to penetrate^18^. Broad-spectrum, non-race-specific resistance based on *mlo* is associated with the formation of callose-containing cell wall appositions called papillae and the secretion of antimicrobial compounds^16^. The papillae constitute a mechanical barrier that prevents penetration by pathogens. The formation of papillae and secretion of antimicrobial compounds depend on the delivery of substances via actin-dependent vesicle transport^27,28^. In addition to susceptibility/resistance to PM disease, MLO proteins are also involved in multiple physiological functions in different tissues, such as root thigmomorphogenesis^29^ and pollen tube reception by the embryo sac in *A. thaliana*^30^. Despite great efforts to uncover the function of MLO proteins, their most critical biochemical function remains elusive.

Calcium ions (Ca^2+^) are essential second messengers, and increases in Ca^2+^ concentration in the cytosol are among the earliest signaling events that occur when plants are challenged by pathogens. In plant-pathogen interactions, the plant immune system is a two-tiered system, consisting of the pathogen-associated molecular pattern (PAMP)-triggered immunity (PTI) and effector-triggered immunity (ETI)^31^. PTI activation can enhance the overall defense ability of plants and protect plants from pathogen attacks^32^, whereas ETI activation usually leads to local programmed cell death, also called the hypersensitivity response, to prevent invasion by pathogens. The Ca^2+^ signature differs between these layers of immunity. For example, PTI activation is associated with Ca^2+^ transients that return to basal levels within minutes^33^, whereas ETI involves an increase in cytosolic Ca^2+^ that lasts for hours^34^. Abscisic acid (ABA), one of the major plant hormones, is also associated with plant defense responses against various pathogens^35,36^. Many studies have shown that ABA regulates defense responses by influencing the deposition of callose, the production of H_2_O_2_, and the expression of defense-related genes^37,38^. Moreover, ABA plays an important role in the regulation of stomatal closure, which acts as a physical barrier for defense against pathogen invasion^39^.

The aim of the present study was to study the function of *CmMLO17* in resistance to *A. alternata*. *CmMLO17* was upregulated after *A. alternata* infection. We cloned the full-length sequence of *CmMLO17* and identified the interacting protein CmKIC, which was also involved in *A. alternata* resistance. RNA sequencing showed that *CmMLO17*-silenced plants had faster and stronger defense responses mediated by ABA and Ca^2+^ signal transduction in the plant-pathogen interaction pathways, resulting in decreased susceptibility of chrysanthemum to *A. alternata* infection.

## Results

### Isolation, sequence analyses, and phylogenetic analysis of CmMLO17

**Fig. 1:**
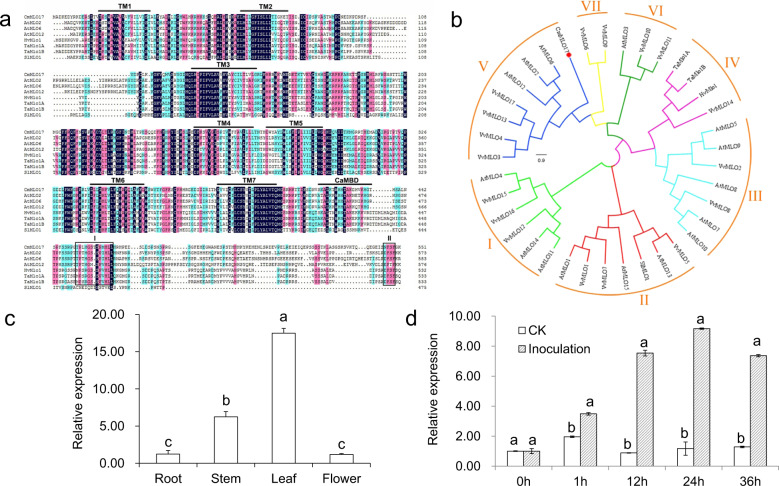
Sequence analysis, phylogenetic tree analysis, and expression pattern analysis of CmMLO17. **a** Sequence alignment of CmMLO17 with other MLO proteins involved in disease susceptibility. The alignment was generated by DNAMAN. The positions of the transmembrane regions (TM1–TM7) and CaMBD are marked with lines above the sequences. The regions of two conserved domains (I and II) at the C-terminus are highlighted in boxes. **b** An unrooted phylogenetic tree was constructed based on MLO sequences from chrysanthemum (CmMLO17) and *A. thaliana*, *Hordeum vulgare*, *Triticum aestivum*, and *Solanum lycopersicum*. The amino acid sequences were aligned using the ClustalW tool in MEGA7 software, and the phylogenetic tree was generated using the neighbor-joining method. The colored arcs represent different clades. The red dot indicates CmMLO17. The sequence details are as follows: AtMLO1 (AT4G02600), AtMLO2 (AT1G11310), AtMLO3 (AT3G45290), AtMLO4 (AT1G11000), AtMLO5 (AT2G33670), AtMLO6 (AT1G61560), AtMLO7 (AT2G17430), AtMLO8 (AT2G17480), AtMLO9 (AT1G42560), AtMLO10 (AT5G65970), AtMLO11 (AT5G53760), AtMLO12 (AT2G39200), AtMLO13 (AT4G24250), AtMLO14 (AT1G26700), AtMLO15 (AT2G44110), VvMLO1 (CAO41068), VvMLO2 (CAO66267), VvMLO3 (CAO18135), VvMLO4 (CAO21819), VvMLO5 (CAO22254), VvMLO6 (CAO66388), VvMLO7 (CAO46388), VvMLO8 (CAO71699), VvMLO9 (CAO84002), VvMLO10 (CAO18134), VvMLO11 (CAO21818), VvMLO12 (CAO39251), VvMLO13 (CAO68971), VvMLO14 (CAO66265), VvMLO15 (CAO47031), VvMLO16 (CAO48195), VvMLO17 (CAO68972), HvMlo1 (CAJW010005773.1), TaMlo1A (AX063298), TaMlo1B (AX063294), SlMLO1 (Solyc01g102520). **c** Expression pattern of *CmMLO17* in the roots, stems, leaves, and flowers of the WT revealed by quantitative real-time PCR (qRT-PCR) with the primers CmMLO17-QRT-F/R (Table S1). **d** Expression pattern of *CmMLO17* in leaves of the WT after *A. alternata* infection revealed via qRT-PCR. CK: Uninfected WT

Chrysanthemum transcriptome libraries were screened using sequences from *Arabidopsis* AtMLO2, AtMLO6, and AtMLO12, and the chrysanthemum *MLO* gene was isolated. Sequence alignment of this gene confirmed that it had high homology with *MLO* genes of other plant species, and it was named *CmMLO17*. Specific primers were designed based on the transcriptome sequences and used to amplify the full-length cDNAs from leaves of wild-type (WT) chrysanthemum ‘Jinba’. The open reading frame (ORF) of CmMLO17 (KJ560361) is 1659 base pairs (bp) in length and encodes 552 amino acids. CmMLO17 is a typical MLO protein, containing seven transmembrane domains and one CaMBD (Fig. 1a). Peptide domain I and a tetrapeptide motif (D/E-F-S/T-F) in region II associated with PM susceptibility^18^ were detected in CmMLO17, albeit with a slight residue change, with the tetrapeptide motif substituted with N-F-S-F in CmMLO17 (Fig. 1a).

Phylogenetic analysis indicated that CmMLO17 is a member of clade V (Fig. 1b), which is mainly related to disease susceptibility^9–11,40,41^. Therefore, we speculate that CmMLO17 may be involved in the response of plants to pathogens.

### The *CmMLO17* gene is highly expressed in leaves and induced by *A. alternata* infection

CmMLO17 is differentially expressed in the root, stem, leaf, and flower tissues of the WT. The transcriptional level of *CmMLO17* was highest in the chrysanthemum leaves, high in stems, and lowest in the roots and flowers (Fig. 1c). At 1 h after *A. alternata* infection, the expression level of *CmMLO17* in the infected plants was approximately two times higher than that in the control. From 12 h to 36 h after invasion by *A. alternata*, the transcription level of CmMLO17 remained significantly greater than that in the uninfected plants (Fig. 1d).

### *CmMLO17* silencing resulted in decreased susceptibility of chrysanthemum to *A. alternata* infection

**Fig. 2:**
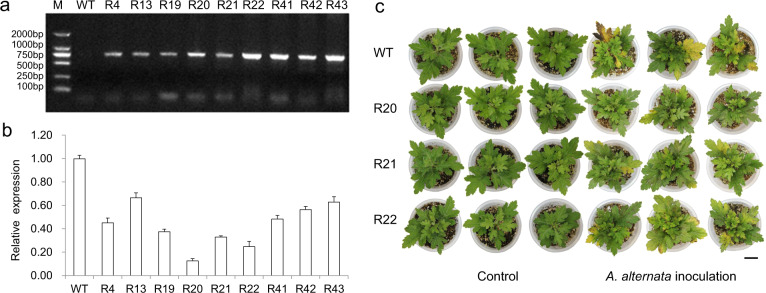
Silencing *CmMLO17* resulted in decreased susceptibility of chrysanthemum to *A. alternata*. **a** Identification of *CmMLO17*-silenced plants by analyzing the *HptII* sequence of the pMDC32-ami*CmMLO17* vector via PCR, in which WT was used as a negative control. R4–R43 lines represent transgenic *CmMLO17-*silenced plants. **b** Relative abundance of *CmMLO17* transcripts in WT and transgenic silenced plants calculated with the 2^−ΔΔCT^ method. **c** Distinct responses of WT and *CmMLO17* transgenic plants to *A. alternata*. Scale bar = 2 cm

Plants with putative CmMLO17 artificial interference were identified using polymerase chain reaction (PCR) based on the *HptII* sequences of the transformed vector (Fig. 2a), and the abundance of CmMLO17 transcripts was analyzed by qRT-PCR (Fig. 2b). Three independent transgenic lines, namely, R20, R21, and R22, that accumulated much lower amounts of *CmMLO17* transcripts were selected for evaluating resistance against *A. alternata*. After inoculation with *A. alternata*, the old leaves in the middle and lower parts of the WT became yellow, and some of the leaves showed black necrotic spots, whereas the leaves of the *CmMLO17*-silenced lines R20, R21, and R22 showed only yellowing at the edge of the leaves, and the degree of yellowing was lower than that in the WT plants (Fig. 2c). The average number of diseased leaves of the WT and *CmMLO17*-silenced lines inoculated with *A. alternata* was counted (Table S2). Only a small part of the leaves of the R20 line, which had the lowest abundance of the *CmMLO17* transcript, appeared slightly yellow, and some of the plants were not diseased. Therefore, transgenic *CmMLO17-*silenced plants were less susceptible to *A. alternata* infection.

### Differentially expressed genes (DEGs) of the calcium and ABA signaling pathways were upregulated in *CmMLO17*-silenced plants

**Fig. 3:**
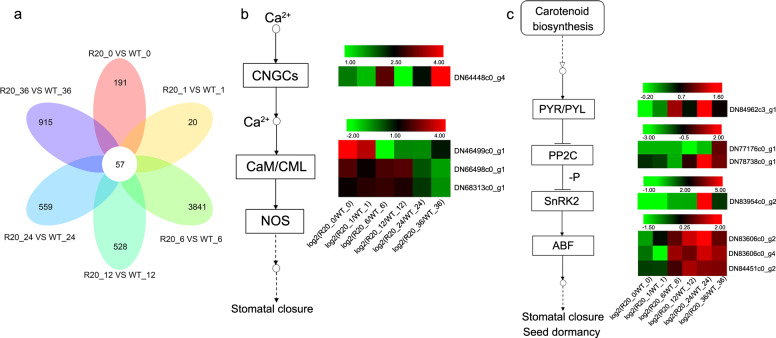
DEGs involved in the Ca^2+^ signaling pathway and ABA signaling pathway were upregulated in the *CmMLO17*-silenced line R20 after infection with *A. alternata*. **a** Venn diagram analysis of DEGs in chrysanthemum leaves. **b** DEGs involved in the Ca^2+^ signaling pathway. **c** DEGs involved in the ABA signaling pathway. Expression values are presented as log_2_ (R20/WT fold change in induced expression). Blocks with colors indicate decreased (green) or increased (red) values. Each column represents the expression values at 0, 1, 6, 12, 24, and 36 hpi from left to right. Each row represents a DEG, with its gene ID shown

To study the variations in gene expression in chrysanthemum leaves after inoculation with *A. alternata*, RNA sequencing was conducted on the WT and a highly resistant line (R20) of *CmMLO17* with silencing at 0, 1, 6, 12, 24, and 36 h post inoculation (hpi). DEGs were identified when their expression levels changed twice or more and the p-adjusted *P* value (padj) was <0.05. The number of DEGs was higher in the comparison between the R20-6 hpi treatment and WT-6 hpi treatment (Fig. 3a). However, the number of DEGs was relatively low in the comparison between the R20-1 hpi treatment and WT-1 hpi treatment. Among the DEGs, many plant defense-related genes were induced, such as calcium and ABA signaling pathway genes. Calcium signaling genes were upregulated during the early and middle stages of *A. alternata* infection, whereas ABA signaling genes showed great changes in transcription levels during the middle and late stages of inoculation (Fig. 3b, c). The calcium and ABA signaling pathways may have contributed to the defense responses against *A. alternata*. Therefore, *CmMLO17*-silenced plants had faster and stronger defense responses, mediated by ABA and Ca^2+^ signaling pathways, than WT plants, resulting in enhanced resistance of chrysanthemum to *A. alternata* infection.

### Identification of DEGs upregulated in *CmMLO17*-silenced plants

**Fig. 4:**
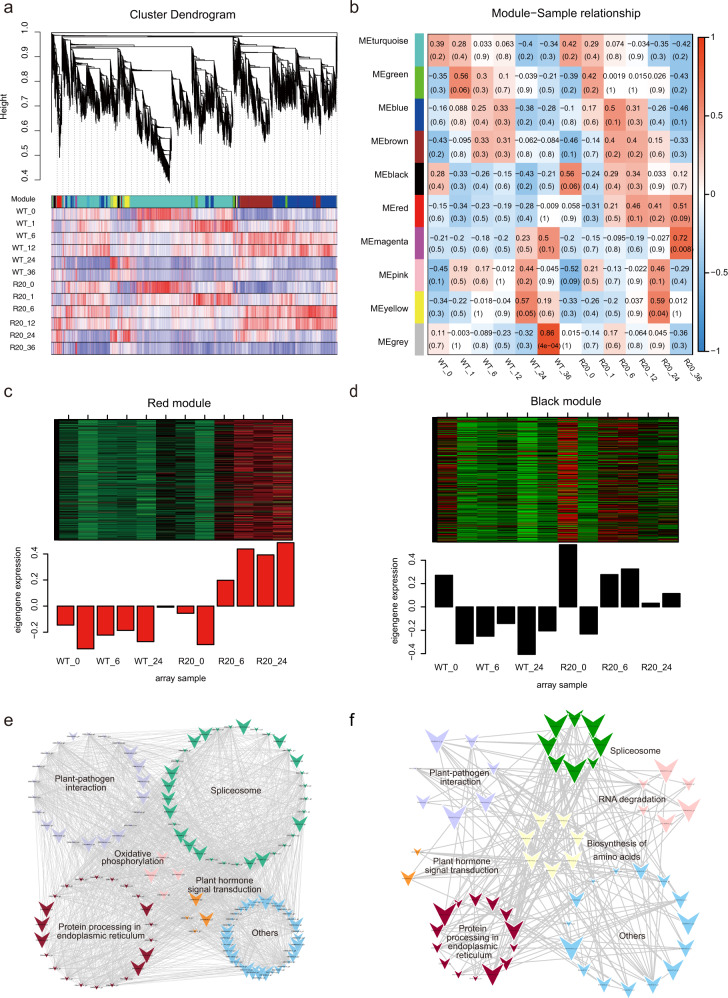
Weighted gene coexpression network analysis revealed a highly expressed gene set in the *CmMLO17*-silenced line R20. **a** Dendrogram of samples based on eigengene expression. **b** Relationship between sample and module. Heatmap of eigengenes in the red (**c**) and black (**d**) modules. Relationship between genes in the red (**e**) and black (**f**) modules, visualized with Cytoscape

Weighted gene coexpression network analysis was performed to identify the genes related to phenotypes and to investigate the coexpression networks to elucidate the difference between WT and *CmMLO17*-silenced plants. Ultimately, ten gene coexpression modules, i.e., ‘turquoise’, ‘green’, ‘blue’, ‘brown’, ‘black’, ‘red’, ‘magenta’, ‘pink’, ‘yellow’, and ‘gray’, were discovered (Fig. 4a, b). The genes from the red and black modules were highly correlated with the traits of each sample group in the *CmMLO17*-silenced plants compared with the WT after *A. alternata* inoculation (Fig. 4b). In the red and black modules, a series of genes were upregulated in *CmMLO17*-silenced plants compared with WT plants (Fig. 4c, d).

Kyoto Encyclopedia of Genes and Genomes (KEGG) annotation analyses and coexpression network analyses were performed to further explore the pathways in which the genes were involved. DEGs in the red module were annotated into multiple KEGG pathways, as shown in Tables S3 and S4. The regulatory network between the multiple signal transduction pathways was visualized using Cytoscape (Fig. 4e, f), which indicated the regulatory relationship among multiple signal transduction pathways. ABA-responsive element binding factor (ABF) homologs were upregulated and were involved in the ABA signal transduction pathway and the plant-pathogen interaction pathway, and 22 genes, including HSF90A and HSF90B homologs, were identified in the red module (Fig. 4e). In the black module, ABF, calcium-dependent protein kinase, and calcium-binding protein were identified as being related to other genes (Fig. 4f) that might play essential roles in the CmMLO17-mediated response to *A. alternata* invasion.

### CmMLO17 interacts with CmKIC in yeast and in planta

**Fig. 5:**
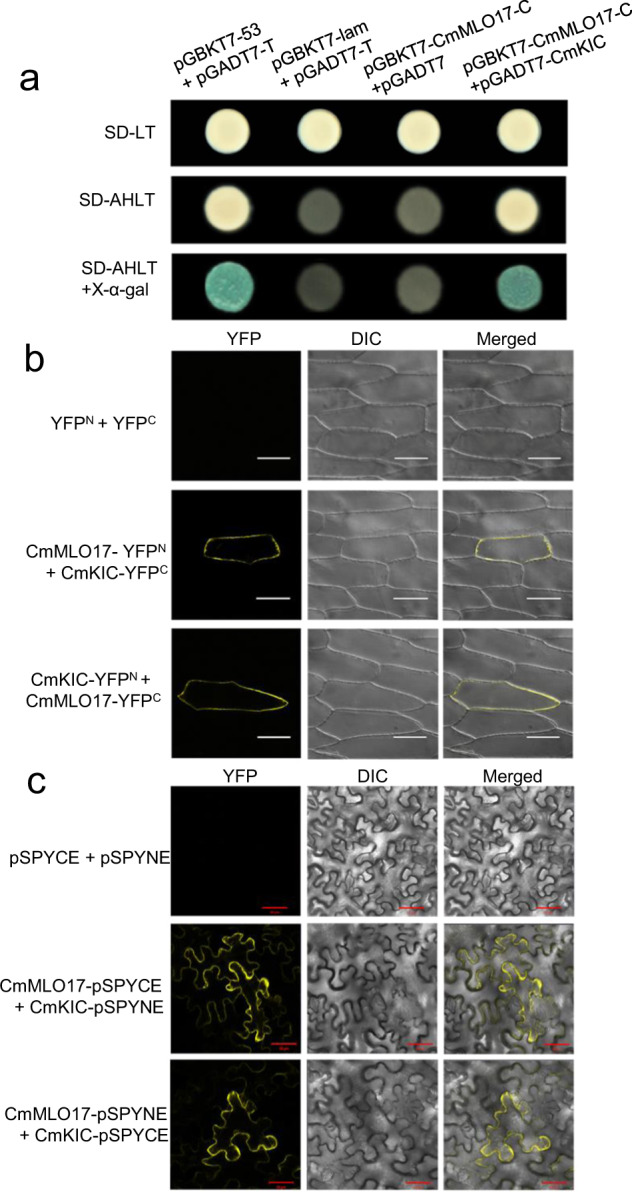
CmMLO17 and CmKIC interact in yeast and plant cells. **a** The C-terminus of CmMLO17 interacts with CmKIC via a yeast two-hybrid system. **b** BiFC analysis of the interaction of CmMLO17 with CmKIC in onion epidermal cells. **c** Interaction assay of CmMLO17 and CmKIC in *N. benthamiana* epidermal cells. YFP yellow fluorescent protein images, DIC visible images, Merged merged fluorescence and visible images. Scale bar = 50 μm

The C-terminus of CmMLO17 was used as bait to screen the cDNA library of *Alternaria*-infected chrysanthemum using the yeast two-hybrid assay, and the candidate gene *KIC* was found. KIC is a Ca^2+^-binding protein with one EF-hand motif that interacts with kinesin-like calmodulin-binding protein (KCBP), which is a microtubule motor protein and plays important roles in the regulation of microtubules^42^. Yeast two-hybrid analysis was performed to determine whether CmMLO17-C interacted with CmKIC. The combinations pGBKT7-53 + pGADT7-T and pGBKT7-Lam + pGADT7-T acted as positive and negative controls, respectively. All yeast colonies with different plasmid combinations grew well on synthetic dropout medium without leucine and tryptophan. The results showed that the yeast colonies transformed with the plasmid combination pGBKT7-*CmMLO17*-C + pGADT7-*CmKIC* grew well on synthetic dropout medium lacking adenine, histidine, leucine, and tryptophan (SD-AHLT) and turned blue on SD-AHLT plates supplemented with X-α-gal, similar to the positive control (Fig. 5a). Yeast cells transformed with the plasmid combination pGBKT7-*CmMLO17*-C + pGADT7 could not grow on SD-AHLT plates, similar to the negative control, suggesting that the C-terminus of CmMLO17 had no autoactivity in yeast cells (Fig. 5a). Together, these results suggest that the C-terminus of CmMLO17 binds with CmKIC in yeast.

Bimolecular fluorescence complementation (BiFC) analysis was performed to further confirm the interaction of CmMLO17 and CmKIC in plant cells (Fig. 5b, c). The pSPYNE and pSPYCE vectors containing YFP^N^ and YFP^C^, respectively, were used to construct CmMLO17-YFP^N^ and CmKIC-YFP^C^, or vice versa. The fusion proteins were transformed into *Agrobacterium tumefaciens* and injected into *Nicotiana benthamiana* leaves, and the fluorescence signals were observed under a scanning confocal microscope. When CmMLO17-YFP^N^ and CmKIC-YFP^C^ were transiently coexpressed, or vice versa, yellow fluorescence was visualized at the plasma membrane, suggesting the interaction of CmMLO17 and CmKIC. No yellow fluorescence was detected upon coexpression of YFP^N^ with YFP^C^ in *N. benthamiana* leaves (Fig. 5c).

We also confirmed the interaction between CmMLO17 and CmKIC in onion epidermal cells. The results of the BiFC analysis revealed that the combinations CmMLO17-YFP^N^ + CmKIC-YFP^C^ and CmMLO17-YFP^C^ + CmKIC-YFP^N^ displayed yellow fluorescence signals at the plasma membrane (Fig. 5b), indicating that CmMLO17 interacted with CmKIC at the plasma membrane.

### Subcellular localization of CmMLO17 and CmKIC

**Fig. 6:**
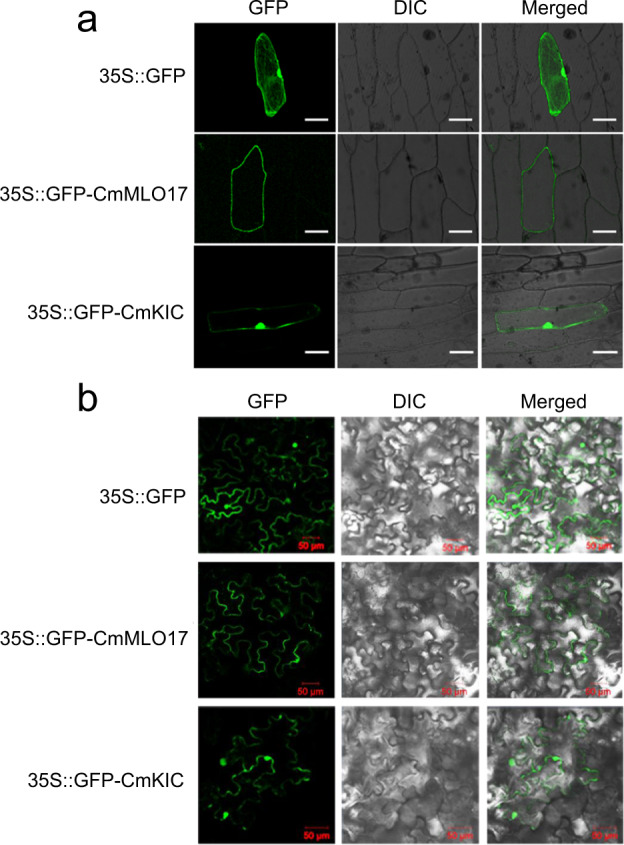
Subcellular localization of CmMLO17 and CmKIC proteins. **a** Subcellular localization of CmMLO17 and CmKIC in onion epidermal cells. **b** Subcellular localization of CmMLO17 and CmKIC in *N. benthamiana* epidermal cells. GFP green fluorescence images, DIC visible images, Merged merged fluorescence and visible images. Scale bar = 50 μm

To analyze the subcellular localization of CmMLO17 and CmKIC, the fusion plasmids 2×35S::*CmMLO17*-GFP, 2×35S::*CmKIC*-GFP, and a positive control, 2×35S::GFP, were separately bombarded into onion epidermal cells. In the cells transformed with 2×35S::GFP, the GFP signals were visualized in the plasma membrane, cytoplasm, and nucleus (Fig. 6a). GFP signals were detected on the plasma membrane of the cells expressing 2×35S::CmMLO17-GFP (Fig. 6a). GFP signals were detected in the plasma membrane and nucleus in cells transformed with 2×35S::CmKIC-GFP (Fig. 6a), indicating that CmKIC localized to both the plasma membrane and nucleus. GFP-tagged CmMLO17 and CmKIC were separately transformed into *Agrobacterium* strain *EHA105* and injected into 5-week-old leaves of *N. benthamiana*. The fluorescence signals were observed using a confocal laser scanning microscope (Fig. 6b), and the results were consistent with the subcellular localization in the onion epidermal cells.

### Overexpression of *CmKIC* increased the sensitivity of chrysanthemum to *A. alternata*, and gene silencing resulted in decreased susceptibility

**Fig. 7:**
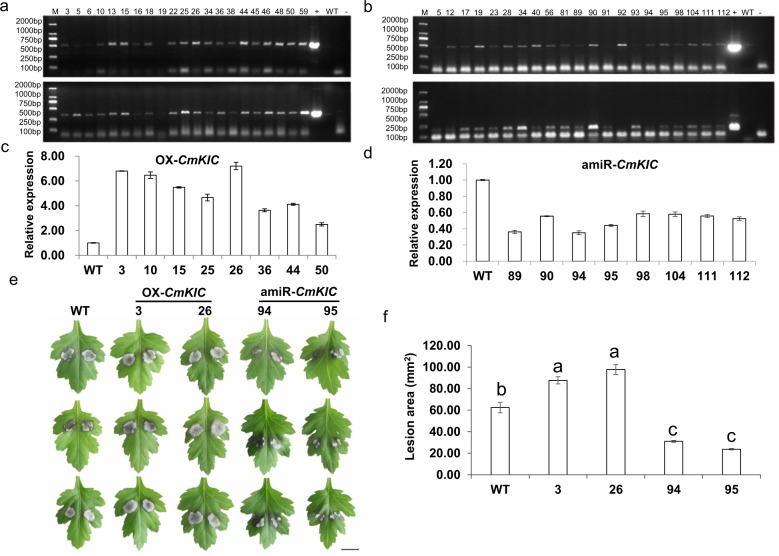
Overexpression of *CmKIC* increased the sensitivity of chrysanthemum to *A. alternata*, and *CmKIC* silencing reduced susceptibility. **a** Identification of *CmKIC*-overexpressing plants. Top: The PCR primers KIC-ID-F1/R1 were used to amplify the region from the nos terminator of the pMDC43 vector to the ORF end of *CmKIC*. Bottom: The PCR primers KIC-ID-F2/R2 were used to detect GFP6 from the vector. **b** Identification of *CmKIC*-silenced plants. Top: The PCR primers KIC-ID-F3/R3 were used to amplify the region from the 35S promoter of the pMDC32 vector to the terminus of amiRNA. Bottom: The PCR primers KIC-ID-F4/R4 were used to detect the amiRNA. +: Positive control. -: Negative control. WT: Wild-type chrysanthemum ‘Jinba’. **c** Relative expression of *CmKIC* in WT and *CmKIC*-overexpressing plants, as determined by qRT-PCR. **d** Relative expression of *CmKIC* in WT and *CmKIC*-silenced plants, as determined by qRT-PCR. **e** Phenotypes of WT and *CmKIC* transgenic plants inoculated with *A. alternata*. Scale bar = 1 cm. **f** Lesion area of WT and *CmKIC* transgenic lines inoculated with *A. alternata*. WT is wild-type chrysanthemum ‘Jinba’, 3 and 26 represent transgenic plants overexpressing *CmKIC*, and 94 and 95 represent transgenic plants with *CmKIC* silenced

Putative transgenic plants were identified by PCR using specific primers (Fig. 7a, b). The relative expression of *CmKIC* in transgenic chrysanthemum was analyzed using qRT-PCR (Fig. 7c, d). The overexpression lines 3 and 26 and the silenced lines 94 and 95 were selected for the *A. alternata* inoculation assay.

A total of 48 hpi with *A. alternata*, the average lesion area was 62.37 mm^2^ on the WT, 87.70 mm^2^ on *CmKIC* overexpression line 3, 97.50 mm^2^ on overexpression line 26, 31.02 mm^2^ on *CmKIC*-silenced line 94 and 30.69 mm^2^ on *CmKIC*-silenced line 95 (Fig. 7e, f). Therefore, overexpression of *CmKIC* increased the sensitivity of chrysanthemum to *A. alternata*, whereas silencing of *CmKIC* significantly reduced the susceptibility to *A. alternata*.

## Discussion

MLO is a family of integral membrane proteins that are highly conserved across the plant kingdom. First discovered in barley, recessive mutants with the loss of function of WT MLO proteins show broad-spectrum resistance to PM fungi^8^. MLO genes have been identified in various monocots and eudicots, including barley, *Arabidopsis*, tomato, and pea^8–10,40^. However, not all MLO genes have been suggested to be related to plant-pathogen interactions. Moreover, the function of MLO in chrysanthemum has not been previously reported. Cloning and determining the functions of the *MLO* gene in chrysanthemum can provide a new direction for breeding disease-resistant chrysanthemum.

The genomic sequence of *C. morifolium* has not been published; therefore, CmMLO17 was isolated based on chrysanthemum transcriptome databases. The expression patterns in leaves inoculated with *A. alternata* showed that CmMLO17 might play a role in the response to *A. alternata*. We chose the artificial microRNA interference approach to knock down *CmMLO17*. In transgenic plants, the expression of *CmMLO17* was significantly reduced (Fig. 2b); however, it was not completely silenced. When inoculated with *A. alternata*, the *CmMLO17*-silenced plants showed less severe symptoms than the WT plants (Fig. 2c). Therefore, *CmMLO17* may have a function similar to that of its orthologs in the MLO clade V subfamily in *A. thaliana*.

Although *mlo* alleles have durable broad-spectrum resistance to PM fungi, they may not function similarly in response to other pathogens. Barley *mlo* plants exhibited increased susceptibility to the hemibiotrophic fungus *Magnaporthe grisea*^43^ and increased sensitivity to toxin-containing culture filtrates of *Bipolaris sorokiniana* compared to WT barley^44^. Furthermore, the susceptibility of the barley *mlo* genotype to Ramularia leaf spot caused by the necrotrophic ascomycete *Ramularia collo-cygni* was apparently affected by environmental conditions^45^. Under field conditions, there seemed to be no difference between *mlo* and WT barley infested with *Fusarium* spp. and *R. collo-cygni*^46^. The barley *mlo* genotype was less sensitive to *Phytophthora palmivora* but only in young leaf tissues^47^. Our study showed that *CmMLO17*-silenced chrysanthemum plants exhibited attenuation of the infection by the necrotrophic fungus *A. alternata*. These data indicate that *mlo* alleles have a trade-off between PM resistance and sensitivity to hemibiotrophic or necrotrophic pathogens, and whether this trade-off is due to pleiotropic phenotypes of *mlo* mutants or environmental conditions still needs further exploration.

When challenged with pathogens, plants produce a series of defense responses to pathogen invasion. When induced by pathogens, different Ca^2+^ sensors, such as CaM, CaM-like proteins (CMLs), Ca^2+^-dependent protein kinases, and calcineurin B-like proteins, can recognize Ca^2+^ signatures and convert the signals to a variety of plant immune responses, consisting of defense-related gene induction and the production of ROS and nitric oxide (NO)^48–51^. The generation of Ca^2+^, ROS, and NO not only induces cell wall reinforcement and a hypersensitive response^48^ but also stimulates stomatal closure in guard cells^52,53^. Several studies have reported that stomata can act as barriers to invasion by pathogens, and PAMP-induced stomatal closure is one of the defense responses adopted by vascular plants to limit pathogenic infection^39^.

In addition to regulating the calcium signaling pathway, ABA is an important mediator of the regulation of stomatal opening and closing^54^. The ABA signaling pathway is mainly composed of three parts, namely, ABA receptor proteins (PYR/PYL/RCAR), type 2C protein phosphatases (PP2C), and SNF1-related protein kinase 2 (SnRK2)^55,56^. Complex formation by PYR/PYL/RCAR and PP2C results in inhibition of the dephosphorylation activity of PP2C, which activates SnRK2 and leads to closure of the stomatal pore. As a physical barrier, stomatal closure can reduce the chance of cell invasion by *A. alternata* hyphae. ABA also plays an important role in regulating stress responses^57^. ABA acts as a positive regulator of the defense against some necrotrophic pathogens, such as *A. brassicicola* and *Plectosphaerella cucumerina*^58^. In our study, the results of the DEG analysis showed that the genes involved in Ca^2+^ and ABA signaling pathways in *CmMLO17*-silenced plants responded faster and more strongly to *A. alternata* infection than the WT plants, thus improving the resistance of transgenic chrysanthemum.

The C-terminus of the MLO protein contains a conserved CaMBD domain, and the CaM protein binds to the CaMBD of MLO in the presence of Ca^2+^ to regulate MLO-dependent disease susceptibility^26^. The interaction between CaMBD at the C-terminus of CaMLO2 and CaCaM1 has been confirmed in pepper; this interaction led to the repression of cell death and defense responses triggered by *Xanthomonas* AvrBsT^41^, suggesting that the C-terminus of MLO may be the region where MLO interacts with other proteins. Therefore, to further explore the function of CmMLO17, the C-terminus of CmMLO17 was used to screen putative interacting proteins. Interestingly, we found the calcium-binding protein KIC.

KIC, which binds and negatively regulates KCBP, represents a novel Ca^2+^-binding protein^42^. KCBP is a member of the kinesin superfamily and plays important roles in the regulation of microtubule organization, microtubule dynamics, and directional vesicle transport^42,59^. Vesicle trafficking in plant-pathogen interactions plays a critical role in secretion-related defense responses, which are an important aspect of *mlo*-based resistance. Barley Ror2 and its ortholog PEN1 in *Arabidopsis* are required molecular components for full resistance of *mlo*^9,60^. Ror2 and PEN1 were found to encode plasma membrane-located syntaxin proteins that are members of the SNARE (soluble N-ethylmaleimide-sensitive factor attachment protein receptor) superfamily and possibly participate in the secretion of antimicrobial compounds at the sites of attempted fungal infection^61^. We hypothesize that KIC, the interacting protein of CmMLO17, binds to KCBP to inhibit the regulation of KCBP in vesicle transport, which may affect secretion-related defense responses and support fungal growth. In this study, the phenotypes of *CmKIC* transgenic plants indirectly confirmed this hypothesis, but direct evidence is needed in further research. Moreover, we observed that the KIC protein is localized in both the plasma membrane and nucleus (Fig. 6), which may be related to its dynamics. In addition to interacting with CmMLO17 on the plasma membrane, CmKIC also binds to transcription factors in the nucleus to regulate defense responses (unpublished). In the future, analysis of biological processes regulated by CmKIC will reveal the biochemical function of MLO proteins.

In conclusion, we found that CmMLO17 interacted with CmKIC at the plasma membrane. Transgenic plants with silencing of *CmMLO17* or *CmKIC* were less susceptible to *A. alternata* infection, indicating that *CmMLO17* and *CmKIC* are involved in pathways that support fungal growth. RNA sequencing showed that ABA and Ca^2+^ signaling pathway genes were altered in *CmMLO17*-silenced plants. Exploring the mechanism by which CmMLO17 and its partner CmKIC are involved in the response of chrysanthemum to *A. alternata* will provide insight into the functions of MLO and defense responses of plants to necrotrophic pathogens.

## Materials and methods

### Plant materials and growth conditions

The WT chrysanthemum cultivar ‘Jinba’ used in this study was obtained from the Chrysanthemum Germplasm Resource Conservation Center, Nanjing Agricultural University, China. The plants were cultivated in a 1:2 (v/v) mixture of soil and vermiculite and were grown in a greenhouse held at 25 °C/22°C in 70% humidity and a 14 h light/10 h dark cycle.

### *A. alternata* inoculation and disease severity assessment

*A. alternata* was cultured on potato dextrose agar medium at 28 °C for 4-5 days. Ten disks (~4 mm in diameter) obtained from the edges were ground in a tissue macerator, transferred to potato dextrose broth medium, and cultured in a shaker at a temperature of 28 °C for 2 days. Mycelia of *A. alternata* were collected from 1 mL of mycelial culture medium and inoculated on the back of the third fully unfolded leaf with a brush. Each leaf had two inoculation points. Each line was inoculated with ten plants, and the experiment was repeated three times independently. The inoculated seedlings were placed in an incubator and cultured in darkness at 25 °C under a relative humidity of 90% for 48 h. Then, the infected area of the inoculated leaves was measured at 2 days post inoculation using ImageJ software. Disease severity was evaluated by the average number of leaves showing disease symptoms and the infected area. Variance analysis was employed to determine the significance of test data according to Tukey’s multiple range test (*P* < 0.05). SPSS v19.0 software was applied for statistical analyses.

### Database searches, cloning, and sequence analysis of CmMLO17

*Arabidopsis* MLO protein sequences of clade V were downloaded from The Arabidopsis Information Resource database and acted as query sequences to identify CmMLO susceptibility genes in chrysanthemum. Homology searches of the MLO sequences were performed, to avoid repetition, with the BLASTX tool at the National Center for Biotechnology Information (NCBI, USA) (http://www.ncbi.nlm.nih.gov). Sequences were fully annotated by taking advantage of prediction programs, including SMART (http://smart.emblheidelberg.de/)^62^, Pfam^63^, NCBI-CDD^64^, and InterProscan (http://www.ebi.ac.uk/interpro/scan.html)^65^. The full ORF sequence of CmMLO17 was amplified using a primer pair (CmMLO17-ORF-F/R) (Table S1), with cDNA from the WT used as a template. The AxyPrep DNA Gel Extraction Kit (Axygen, China) was used to purify the putative amplicons, and the purified fragments were ligated into the pMD19-T (Takara, Japan) vector for sequencing.

To study the evolutionary relationships, an unrooted neighbor-joining phylogenetic tree of CmMLO17 with its homologs in several other species was constructed using the MEGA7 software program^66^, and a bootstrap test with 1000 replicates was performed. The DNAMAN and ClustalW software programs were used to align the homology of MLO peptide sequences.

### Yeast two-hybrid screening

For yeast two-hybrid screening, the C-terminal subclone of CmMLO17 (amino acids 405 to 552), including the CaMBD, was amplified by PCR from a full-length CmMLO17 clone in pMD19-T using the primers BD-CmMLO17-C-F and BD-CmMLO17-C-R (for specific primers, refer to Table S1). After purification, the amplified PCR product and pGBKT7 empty plasmid were digested by *Eco*R I and *Sal* I, respectively, and were then ligated. The pGBKT7-CmMLO17-C plasmid was verified by sequencing and acted as bait to screen the interaction library of chrysanthemum and *Alternaria* in the pGADT7 vector. Transformation of the Y2H yeast strain was performed according to the Matchmaker Gold Yeast Two-Hybrid Kit (Clontech, Mountain View, CA, USA). Putative positive clones were obtained and sequenced. The BLAST program was used to search the homologous genes of the obtained sequences, and SMART and InterProscan were applied to predict the domains of the sequences. The results showed that the KIC protein was the most frequently targeted. Interaction assays in yeast were performed using plasmids carrying pGADT7-*CmKIC* and pGBKT7-*CmMLO17*-C.

### Bimolecular fluorescence complementation analysis

Genes encoding the tested proteins were cloned into the multiple cloning sites of different pSAT4A BiFC vectors^67^ as follows. The ORFs of *CmMLO17* and *CmKIC* were PCR-amplified using the primers BiFC-MLO17-F1/BiFC-MLO17-R1 and BiFC-KIC-F1/BiFC-KIC-R1 (Table S1) with the restriction sites of *Eco*R I and *Sma* I. The amplicons were subsequently digested using *Eco*R I and *Sma* I, and ligated into pSAT4A-cEYFP/nEYFP-N1 vectors digested with the same enzymes, generating pSAT4A-nEYFP/cEYFP-*CmMLO17* and pSAT4A-cEYFP/nEYFP-*CmKIC*, respectively. For the transformation experiments, the mixing of plasmids encoding cEYFP and nEYFP fusion proteins and microbombardment into onion epidermal cells were performed as previously described^67^.

To further confirm the interaction between CmMLO17 and CmKIC, we constructed BiFC vectors for the tobacco system. The ORFs of *CmMLO17* and *CmKIC* were cloned using BiFC-MLO17-F2/BiFC-MLO17-R2 and BiFC-KIC-F2/BiFC-KIC-R2 primers (Table S1) via *Xba* I/*Kpn* I into the vectors pSPYNE173 and pSPYCE(M)^68^, generating *CmMLO17-*pSPYCE/pSPYNE and *CmKIC-*pSPYNE/pSPYCE, respectively. For transient transformation, the GV3101 strain of *A. tumefaciens* carrying the BiFC constructs and p19 strain were used for infiltration of 5-week-old *N. benthamiana* leaves. Infiltration experiments and microscopic analyses were performed as previously described^68^.

### Subcellular localization

The coding region of *CmKIC* was PCR-amplified, with pGADT7-*CmKIC* used as a template. According to the multiple cloning sites, the ORF sequences of *CmMLO17* and *CmKIC* were ligated into the vector pENTR1A (Invitrogen, USA) to generate the vectors pENTR1A-*CmMLO17* and pENTR1A-*CmKIC*, using the *Bam*H I/*Sal* I and *Not* I restriction enzymes, respectively. LR Clonase II enzyme mix (Invitrogen, USA) was used to recombine pENTR1A-CmMLO17 or pENTR1A-CmKIC with pMDC43-GFP to construct GFP-CmMLO17 and GFP-CmKIC fusion vectors driven by the 2×35S promoter. A particle gun (PDS-1000; Bio-Rad, USA) was used to bombard the plasmids pMDC43-*CmMLO17* and pMDC43-*CmKIC* into onion epidermal cells^69^. We also transferred the constructed fusion plasmids into the *Agrobacterium* strain *EHA105* and then injected the leaves of *N. benthamiana* to observe the localization of CmMLO17 and CmKIC. The GFP signals in the transformed cells were detected under a laser scanning confocal microscope (Leica, Germany).

### Constructs of amiRNAi

Sequences of CmMLO17 and CmKIC were submitted to the artificial microRNA design program WMD (http://wmd3.weigelworld.org/) to design the candidate microRNAs. The six specific sequences were designed (I to IV, A and B; listed in Table S1) for engineering the artificial microRNAs through site-directed mutagenesis, for which the plasmid pRS300 containing the precursor of miR319a was used as a template. The amiRNA was constructed using overlap PCR as previously reported^70^. The PCR amplicons were digested with *Sal* I and *Not* I and inserted into the pENTR1A vector digested with the same enzymes. For plant transformation, pENTR1A-ami*CmMLO17* and pENTR1A-ami*CmKIC* were digested with *Nsi* I and introduced into the pMDC32 vector by LR recombination.

### Generation of transgenic chrysanthemum plants and molecular analysis

To clarify the function of *CmMLO17* and *CmKIC*, the overexpression vector of *CmMLO17* and overexpression and silencing vectors of *CmKIC* were transferred into the *EHA105* strain of *A. tumefaciens* using the freeze-thaw method. Chrysanthemum plants were transformed using *Agrobacterium*-mediated methods as previously described^71^, and hygromycin was used to select putative transgenic plants. DNA was extracted from the WT and transformants and used in PCR with specific primers (Table S1) to identify successfully transformed plants. The primers Hyg-F/R were used to detect the pMDC32-ami*CmMLO17* vector in transformation lines. The primers KIC-ID-F1/R1 and KIC-ID-F2/R2 were used to amplify the pMDC43-*CmKIC* vector fragment. The primers KIC-ID-F3/R3 and KIC-ID-F4/R4 were used for amplification of the pMDC32-ami*CmKIC* vector. RNA was extracted and used in qPCR experiments with the primers CmMLO17-QRT-F/CmMLO17-QRT-R and KIC-QRT-F/KIC-QRT-R (Table S1) to measure the expression levels of *CmMLO17* and *CmKIC*, respectively.

### Gene expression analysis by qRT-PCR

Tissues of roots, stems, leaves, and flowers were sampled from the WT to elucidate the expression patterns of *CmMLO17* in different tissues. To determine whether *CmMLO17* was induced after *A. alternata* infection, leaves were sampled before infection and then 1, 12, 24, and 36 hpi. To analyze the relative expression of *CmMLO17* and *CmKIC*, leaves from the same part of the WT and transgenic plants were harvested.

According to the manufacturer’s instructions, RNA was extracted using RNAiso reagent (TaKaRa) and digested with RNase-free DNase I (TaKaRa) to remove genomic DNA. Reverse transcription was performed using a Reverse Transcription Kit (TaKaRa). The transcript levels of *CmMLO17* and *CmKIC* were detected with qRT-PCR assays using SYBR Premix Ex Taq II from the same company as mentioned above following the manufacturer’s instructions. The CmMLO17-QRT-F/CmMLO17-QRT-R and KIC-QRT-F/KIC-QRT-R primer pairs (Table S1) were designed using Primer Express 3.0.1 software, and the *EF1α* gene was used as a reference. A Roche Lightcycler 480 (Roche, Switzerland) was used to perform qRT-PCR. Three biological replicates were performed for each sample. The qRT-PCR data were analyzed by the 2^−ΔΔCT^ method as previously described^72^.

### RNA sequencing and DEG identification analysis

After inoculation with *A. alternata*, the treated leaves were harvested at 0, 1, 6, 12, 24, and 36 hpi and used for RNA sequencing, with three replicates at each time point. Total RNA from the 36 samples was extracted as mentioned above. The libraries of all samples were constructed and sequenced at the Novogene Bioinformatics Institute (Tianjin, China). High-quality reads (clean reads) were extracted from the raw reads after eliminating low-quality reads and filtering adapter sequences. The clean reads were reassembled using Trinity software and matched using RSEM software to acquire the chrysanthemum unigenes of the transcriptome. DEGs were identified using DESeq. To define the DEGs, the criteria for absolute values of log_2_ (induction of expression of transgenic plants compared with WT at defined time points) >1 and padj <0.05 were met. To study the function of the DEGs, various databases were used for gene annotation, such as the clusters of orthologous groups of proteins, gene ontology, KEGG, NCBI nonredundant protein sequence, NCBI nucleotide sequence, protein family, and Swiss-Prot databases. Weighted gene coexpression network analysis was performed with the R package for the identified modules^73,74^. Cytoscape software was used to visualize the coexpression networks^75^.

## Supplementary information

Supplementary information

## References

[1] Dodds PN, Rathjen JP (2010). Plant immunity: towards an integrated view of plant-pathogen interactions. Nat. Rev. Genet..

[2] Morel JB, Dangl JL (1997). The hypersensitive response and the induction of cell death in plants. Cell Death Differ..

[3] Nimchuk Z, Eulgem T, Holt BF, Dangl JL (2003). Recognition and response in the plant immune system. Annu Rev. Genet..

[4] Collier SM, Moffett P (2009). NB-LRRs work a “bait and switch” on pathogens. Trends Plant Sci..

[5] Le Roux C (2015). A receptor pair with an integrated decoy converts pathogen disabling of transcription factors to immunity. Cell.

[6] Parlevliet, J. E. What is durable resistance, a general outline. in *Durability of Disease Resistance*. 23–39 (Springer, Dordrecht, 1993).

[7] Pavan, S., Jacobsen, E., Visser, R. G. & Bai, Y. Loss of susceptibility as a novel breeding strategy for durable and broad-spectrum resistance. *Mol. Breed* **25**, 1–12 (2010). 10.1007/s11032-009-9323-6 PMC2837247 20234841

[8] Jørgensen IH (1992). Discovery, characterization and exploitation of Mlo powdery mildew resistance in barley. Euphytica.

[9] Consonni C (2006). Conserved requirement for a plant host cell protein in powdery mildew pathogenesis. Nat. Genet..

[10] Bai Y (2008). Naturally occurring broad-spectrum powdery mildew resistance in a central American tomato accession is caused by loss of Mlo function. Mol. Plant Microbe Interact..

[11] Pavan S (2011). Pea powdery mildew *er1* resistance is associated to loss-of-function mutations at a *MLO* homologous locus. Theor. Appl. Genet..

[12] Zheng Z (2013). Loss of function in Mlo orthologs reduces susceptibility of pepper and tomato to powdery mildew disease caused by Leveillula taurica. PLoS ONE.

[13] Wang Y (2014). Simultaneous editing of three homoeoalleles in hexaploid bread wheat confers heritable resistance to powdery mildew. Nat. Biotechnol..

[14] Pessina S (2016). The knock-down of the expression of *MdMLO19* reduces susceptibility to powdery mildew (*Podosphaera leucotricha*) in apple (*Malus domestica*). Plant Biotechnol. J..

[15] Pessina S (2016). Knockdown of MLO genes reduces susceptibility to powdery mildew in grapevine. Hortic. Res.

[16] Acevedo-Garcia J, Kusch S, Panstruga R (2014). Magical mystery tour: MLO proteins in plant immunity and beyond. N. Phytol..

[17] Kusch S, Pesch L, Panstruga R (2016). Comprehensive phylogenetic analysis sheds light on the diversity and origin of the MLO Family of integral membrane proteins. Genome Biol. Evol..

[18] Panstruga R (2005). Serpentine plant MLO proteins as entry portals for powdery mildew fungi. Biochem Soc. Trans..

[19] Reinstadler A, Muller J, Czembor JH, Piffanelli P, Panstruga R (2010). Novel induced mlo mutant alleles in combination with site-directed mutagenesis reveal functionally important domains in the heptahelical barley Mlo protein. BMC Plant Biol..

[20] Feechan A, Jermakow AM, Torregrosa L, Panstruga R, Dry IB (2008). Identification of grapevine *MLO* gene candidates involved in susceptibility to powdery mildew. Funct. Plant Biol..

[21] Winterhagen P, Howard SF, Qiu W, Kovács LG (2008). Transcriptional up-regulation of grapevine *MLO* genes in response to powdery mildew infection. Am. J. Enol. Vitic..

[22] Piffanelli P (2002). The barley MLO modulator of defense and cell death is responsive to biotic and abiotic stress stimuli. Plant Physiol..

[23] Qiu X (2015). Antisense RhMLO1 gene transformation enhances resistance to the Powdery Mildew pathogen in *Rosa multiflora*. Plant Mol. Biol. Rep..

[24] Devoto A (1999). Topology, subcellular localization, and sequence diversity of the Mlo family in plants. J. Biol. Chem..

[25] Kim MC (2002). Mlo, a modulator of plant defense and cell death, is a novel calmodulin-binding protein. Isolation and characterization of a rice Mlo homologue. J. Biol. Chem..

[26] Kim MC (2002). Calmodulin interacts with MLO protein to regulate defence against mildew in barley. Nature.

[27] Miklis M (2007). Barley MLO modulates actin-dependent and actin-independent antifungal defense pathways at the cell periphery. Plant Physiol..

[28] Feechan A, Kabbara S, Dry IB (2011). Mechanisms of powdery mildew resistance in the Vitaceae family. Mol. Plant Pathol..

[29] Bidzinski P (2014). Physiological characterization and genetic modifiers of aberrant root thigmomorphogenesis in mutants of *Arabidopsis thaliana MILDEW LOCUS O* genes. Plant Cell Environ..

[30] Kessler SA (2010). Conserved molecular components for pollen tube reception and fungal invasion. Science.

[31] Jones JD, Dangl JL (2006). The plant immune system. Nature.

[32] Zipfel C (2004). Bacterial disease resistance in Arabidopsis through flagellin perception. Nature.

[33] Lecourieux D (2005). Proteinaceous and oligosaccharidic elicitors induce different calcium signatures in the nucleus of tobacco cells. Cell Calcium.

[34] Grant M (2000). The RPM1 plant disease resistance gene facilitates a rapid and sustained increase in cytosolic calcium that is necessary for the oxidative burst and hypersensitive cell death. Plant J..

[35] Bari R, Jones JD (2009). Role of plant hormones in plant defence responses. Plant Mol. Biol..

[36] Mauch-Mani B, Mauch F (2005). The role of abscisic acid in plant-pathogen interactions. Curr. Opin. Plant Biol..

[37] Flors V (2008). Interplay between JA, SA and ABA signalling during basal and induced resistance against *Pseudomonas syringae* and *Alternaria brassicicola*. Plant J..

[38] Xing Y, Jia W, Zhang J (2008). AtMKK1 mediates ABA-induced CAT1 expression and H_2_O_2_ production via AtMPK6-coupled signaling in Arabidopsis. Plant J..

[39] Melotto M, Underwood W, Koczan J, Nomura K, He SY (2006). Plant stomata function in innate immunity against bacterial invasion. Cell.

[40] Humphry M, Reinstadler A, Ivanov S, Bisseling T, Panstruga R (2011). Durable broad-spectrum powdery mildew resistance in pea er1 plants is conferred by natural loss-of-function mutations in *PsMLO1*. Mol. Plant Pathol..

[41] Kim DS, Choi HW, Hwang BK (2014). Pepper *mildew resistance locus O* interacts with pepper calmodulin and suppresses Xanthomonas AvrBsT-triggered cell death and defense responses. Planta.

[42] Reddy VS, Day IS, Thomas T, Reddy AS (2004). KIC, a novel Ca^2+^ binding protein with one EF-Hand motif, interacts with a microtubule motor protein and regulates trichome morphogenesis. Plant Cell.

[43] Jarosch B, Kogel KH, Schaffrath U (1999). The ambivalence of the barley Mlo locus: mutations conferring resistance against powdery mildew (*Blumeria graminis* f. sp. *hordei*) enhance susceptibility to the rice blast fungus *Magnaporthe grisea*. Mol. Plant Microbe Interact..

[44] Kumar J, Hückelhoven R, Beckhove U, Nagarajan S, Kogel KH (2001). A compromised Mlo pathway affects the response of barley to the necrotrophic fungus *Bipolaris sorokiniana* (Teleomorph: *Cochliobolus sativus*) and its toxins. Phytopathology.

[45] McGrann GRD (2014). A trade off between *mlo* resistance to powdery mildew and increased susceptibility of barley to a newly important disease, Ramularia leaf spot. J. Exp. Bot..

[46] Hofer K, Linkmeyer A, Textor K, Hückelhoven R, Hess M (2015). *MILDEW LOCUS O* mutation does not affect resistance to grain infections with Fusarium spp. and *Ramularia collo-cygni*. Phytopathology.

[47] Le Fevre R, O’Boyle B, Moscou MJ, Schornack S (2016). Colonization of Barley by the broad-host hemibiotrophic pathogen *Phytophthora palmivora* uncovers a leaf development-dependent involvement of Mlo. Mol. Plant Microbe Interact..

[48] Poovaiah BW, Du L, Wang H, Yang T (2013). Recent advances in calcium/calmodulin-mediated signaling with an emphasis on plant-microbe interactions. Plant Physiol..

[49] Seybold H (2014). Ca^2+^ signalling in plant immune response: from pattern recognition receptors to Ca^2+^ decoding mechanisms. N. Phytol..

[50] Yuan P, Jauregui E, Du L, Tanaka K, Poovaiah BW (2017). Calcium signatures and signaling events orchestrate plant-microbe interactions. Curr. Opin. Plant Biol..

[51] Zipfel C, Oldroyd GE (2017). Plant signalling in symbiosis and immunity. Nature.

[52] Ward JM, Schroeder JI (1994). Calcium-activated K.^+^ channels and calcium-induced calcium release by slow vacuolar ion channels in guard cell vacuoles implicated in the control of stomatal closure. Plant Cell.

[53] Sun L (2017). NADK2 positively modulates abscisic acid-induced stomatal closure by affecting accumulation of H_2_O_2_, Ca^2+^ and nitric oxide in Arabidopsis guard cells. Plant Sci..

[54] Cai S (2017). Evolutionary conservation of ABA signaling for stomatal closure. Plant Physiol..

[55] Park SY (2009). Abscisic acid inhibits type 2C protein phosphatases via the PYR/PYL family of START proteins. Science.

[56] Ma Y (2009). Regulators of PP2C phosphatase activity function as abscisic acid sensors. Science.

[57] Wasilewska A (2008). An update on abscisic acid signaling in plants and more. Mol. Plant.

[58] Ton J, Mauch-Mani B (2004). Beta-amino-butyric acid-induced resistance against necrotrophic pathogens is based on ABA-dependent priming for callose. Plant J..

[59] Yamada M, Tanaka-Takiguchi Y, Hayashi M, Nishina M, Goshima G (2017). Multiple kinesin-14 family members drive microtubule minus end-directed transport in plant cells. J. Cell Biol..

[60] Freialdenhoven A (1994). *Nar-1* and *Nar-2*, two loci required for *Mla12*-specified race-specific resistance to powdery mildew in barley. Plant Cell.

[61] Collins NC (2003). SNARE protein-mediated disease resistance at the plant cell wall. Nature.

[62] Schultz J, Milpetz F, Bork P, Ponting CP (1998). SMART, a simple modular architecture research tool: identification of signalling domains. Proc. Natl Acad. Sci. USA.

[63] El-Gebali S (2019). The Pfam protein families database in 2019. Nucleic Acids Res..

[64] Marchler-Bauer A (2017). CDD/SPARCLE: functional classification of proteins via subfamily domain architectures. Nucleic Acids Res..

[65] Quevillon E (2005). InterProScan: protein domains identifier. Nucleic Acids Res..

[66] Kumar S, Stecher G, Tamura K (2016). MEGA7: molecular evolutionary genetics analysis version 7.0 for bigger datasets. Mol. Biol. Evol..

[67] Citovsky V (2006). Subcellular localization of interacting proteins by bimolecular fluorescence complementation in planta. J. Mol. Biol..

[68] Waadt R (2008). Multicolor bimolecular fluorescence complementation reveals simultaneous formation of alternative CBL/CIPK complexes in planta. Plant J..

[69] von Arnim, A. Subcellular localization of GUS- and GFP-tagged proteins in onion epidermal cells. *CSH Protoc* 2007, pdb.prot4689, 10.1101/pdb.prot4689 (2007). 10.1101/pdb.prot4689 21357024

[70] Schwab R, Ossowski S, Riester M, Warthmann N, Weigel D (2006). Highly specific gene silencing by artificial microRNAs in Arabidopsis. Plant Cell.

[71] Li P (2015). The over-expression of a chrysanthemum WRKY transcription factor enhances aphid resistance. Plant Physiol. Biochem..

[72] Livak KJ, Schmittgen TD (2001). Analysis of relative gene expression data using real-time quantitative PCR and the 2(-Delta Delta C(T)) method. Methods.

[73] Stuart JM, Segal E, Koller D, Kim SK (2003). A gene-coexpression network for global discovery of conserved genetic modules. Science.

[74] Zhang, B. & Horvath, S. A general framework for weighted gene co-expression network analysis. *Stat Appl Genet Mol Biol* **4**, Article17, 10.2202/1544-6115.1128 (2005). 10.2202/1544-6115.1128 16646834

[75] Shannon P (2003). Cytoscape: a software environment for integrated models of biomolecular interaction networks. Genome Res..

