# Supplementary material for: CmMLO17 and its partner CmKIC potentially support Alternaria alternata growth in Chrysanthemum morifolium
Source: Hortic Res. 2021 May 1;8:101. doi: 10.1038/s41438-021-00534-x (PMC8087703; doi:10.1038/s41438-021-00534-x)
Supplement: Supplementary file 1 — Supplementary information [file 41438_2021_534_MOESM1_ESM.doc]

**Table S1 Primer sequences used in this study**

| Primer | Sequence (5′-3′) | Relevant characteristics |
| --- | --- | --- |
| CmMLO17-ORF-F | AGATACCTAAGAGCACAGATACAAT | PCR primers used to amplify the ORF of CmMLO17 |
| CmMLO17-ORF-R | TTGGTACATTAAGTTCAAGTAGTTT |
| BD-CmMLO17-C-F | GGCCGAATTCACACAGATGGGATCAAGAATGA | PCR primers for amplification of pGBKT7-CmMLO17 for vector construction |
| BD-CmMLO17-C-R | GCAGGTCGACGACTTTTTCCAAATGAAAAATTAG |
| AD-KIC-F | CAGTGAATTCATGGAGGAAGAAAAACAATA | PCR primers for amplification of pGADT7-CmKIC for vector construction |
| AD-KIC-R | CGATGGATCCCTATATTATCCAACGTCGAAG |
| BiFC-MLO17-F1 | CTTCGAATTCATGGCCGATAAAGAAGATTA | PCR primers for amplification of CmMLO17-YFP for vector construction |
| BiFC-MLO17-R1 | GGATCCCGGGACTTTTTCCAAATGAAAAAT |
| BiFC-MLO17-F2 | GCTCTAGAATGGCCGATAAAGAAGATTATAGG | PCR primers for amplification of CmMLO17-pSPYCE/pSPYNE for vector construction |
| BiFC-MLO17-R2 | GGGGTACCACTTTTTCCAAATGAAAAATTAGAAATTTCACCC |
| BiFC-KIC-F1 | CTTCGAATTCATGGAGGAAGAAAAACAATA | PCR primers for amplification of CmKIC-YFP for vector construction |
| BiFC-KIC-R1 | GGATCCCGGGTATATTATCCAACGTCGAAG |
| BiFC-KIC-F2 | GCTCTAGAATGGAGGAAGAAAAACAATACC | PCR primers for amplification of CmKIC-pSPYCE/pSPYNE for vector construction |
| BiFC-KIC-R2 | GGGGTACCTATATTATCCAACGTCGAAGAAG |
| Hyg-F | CTTCTACACAGCCATCGGTCCAG | PCR primers for amplification of pMDC32-ami*CmMLO17* vector in transformation lines |
| Hyg-R | CGGAAGTGCTTGACATTGGGGAG |
| EF1α-F | TTTTGGTATCTGGTCCTGGAG | PCR primers for amplification of the reference gene Cm*EF1α* in quantitative real-time PCR assays |
| EF1α-R | CCATTCAAGCGACAGACTCA |
| CmMLO17-QRT-F | AAAGACGGTTGAATACCAGTACCAT | PCR primers for amplification of *CmMLO17* in quantitative real-time PCR assays |
| CmMLO17-QRT-R | CTTGTTGGCTCTCGGGAGTTAAATGTGTA |
| MLO17-I | GATGTCTTAACTGATTTCGACCGTCTCTCTTTTGTATTCC | PCR primers for amplification of *CmMLO17* microRNA for vector construction |
| MLO17-II | GACGGTCGAAATCAGTTAAGACATCAAAGAGAATCAATGA |
| MLO17-III | GACGATCGAAATCAGATAAGACTTCACAGGTCGTGATATG |
| MLO17-IV | GAAGTCTTATCTGATTTCGATCGTCTACATATATATTCCT |
| Oligo A | CTGCAAGGCGATTAAGTTGGGTAAC | Universal PCR primers for amplification of microRNA for vector construction |
| Oligo B | GCGGATAACAATTTCACACAGGAAACAG |
| KIC-I | GATAACGTGTTTACGTCTAGCCTTCTCTCTTTTGTATTCC | PCR primers for amplification of *CmKIC* microRNA for vector construction |
| KIC-II | GAAGGCTAGACGTAAACACGTTATCAAAGAGAATCAATGA |
| KIC-III | GAAGACTAGACGTAATCACGTTTTCACAGGTCGTGATATG |
| KIC-IV | GAAAACGTGATTACGTCTAGTCTTCTACATATATATTCCT |
| KIC-ID-F1 | ATGGAGGAAGAAAAACAATACCAAG | PCR primers for amplification of pMDC43-*CmKIC* vector in transformation lines |
| KIC-ID-R1 | ACCCATCTCATAAATAAC |
| KIC-ID-F2 | CTGTCCACACAATCTGCC | PCR primers for amplification of pMDC43-*CmKIC* vector in transformation lines |
| KIC-ID-R2 | TATATTATCCAACGTCGAAGAAG |
| KIC-ID-F3 | GACGCACAATCCCACTATCC | PCR primers for amplification of pMDC32-ami*CmKIC* vector in transformation lines |
| KIC-ID-R3 | GAAGGCTAGACGTAAACACGTTATCAAAGAGAATCAATGA |
| KIC-ID-F4 | GAAGACTAGACGTAATCACGTTTTCACAGGTCGTGATATG | PCR primers for amplification of pMDC32-ami*CmKIC* vector in transformation lines |
| KIC-ID-R4 | GAAGGCTAGACGTAAACACGTTATCAAAGAGAATCAATGA |
| KIC-QRT-F | GCTAGCGGACCAGGAAAGTG | PCR primers for amplification of *CmKIC* in quantitative real-time PCR assays |
| KIC-QRT-R | TCATTCCTTCCATACCCAAAATTC |

***Table S2 Average number of diseased leaves of WT and CmMLO17-silenced lines inoculated with A. alternata***

| Materials | Average number of diseased leaves per plant |
| --- | --- |
| WT | 2.04 ± 0.40 a |
| R20 | 0.94 ± 0.24 c |
| R21 | 1.26 ± 0.51 b |
| R22 | 1.34 ± 0.33 b |

**Table S3 KEGG annotation of genes in the red module**

| **Gene ID** | **1st-level (pathway)** | **2nd-level (pathway)** | **3rd-level (pathway)** | **Pathway id** | **KO id** | **KO Description** |
| --- | --- | --- | --- | --- | --- | --- |
| DN63694c0_g3 | Cellular Processes | Transport and catabolism | Endocytosis | ko04144 | K03283 | heat shock 70kDa protein 1/8 |
| DN72862c2_g3 | Cellular Processes | Transport and catabolism | Endocytosis | ko04144 | K03283 | heat shock 70kDa protein 1/8 |
| DN58604c2_g2 | Cellular Processes | Transport and catabolism | Endocytosis | ko04144 | K03283 | heat shock 70kDa protein 1/8 |
| DN63694c0_g2 | Cellular Processes | Transport and catabolism | Endocytosis | ko04144 | K03283 | heat shock 70kDa protein 1/8 |
| DN77812c1_g1 | Cellular Processes | Transport and catabolism | Endocytosis | ko04144 | K03283 | heat shock 70kDa protein 1/8 |
| DN72862c2_g1 | Cellular Processes | Transport and catabolism | Endocytosis | ko04144 | K03283 | heat shock 70kDa protein 1/8 |
| DN70478c3_g1 | Cellular Processes | Transport and catabolism | Endocytosis | ko04144 | K03283 | heat shock 70kDa protein 1/8 |
| DN63724c1_g1 | Cellular Processes | Transport and catabolism | Endocytosis | ko04144 | K03283 | heat shock 70kDa protein 1/8 |
| DN63694c0_g1 | Cellular Processes | Transport and catabolism | Endocytosis | ko04144 | K03283 | heat shock 70kDa protein 1/8 |
| DN76620c0_g2 | Cellular Processes | Transport and catabolism | Endocytosis | ko04144 | K03283 | heat shock 70kDa protein 1/8 |
| DN77142c1_g1 | Cellular Processes | Transport and catabolism | Endocytosis | ko04144 | K03283 | heat shock 70kDa protein 1/8 |
| DN70992c0_g1 | Cellular Processes | Transport and catabolism | Endocytosis | ko04144 | K03283 | heat shock 70kDa protein 1/8 |
| DN77142c2_g2 | Cellular Processes | Transport and catabolism | Endocytosis | ko04144 | K03283 | heat shock 70kDa protein 1/8 |
| DN81155c1_g6 | Cellular Processes | Transport and catabolism | Endocytosis | ko04144 | K03283 | heat shock 70kDa protein 1/8 |
| DN56797c0_g1 | Cellular Processes | Transport and catabolism | Endocytosis | ko04144 | K03283 | heat shock 70kDa protein 1/8 |
| DN65094c0_g1 | Cellular Processes | Transport and catabolism | Endocytosis | ko04144 | K03283 | heat shock 70kDa protein 1/8 |
| DN95710c3_g2 | Cellular Processes | Transport and catabolism | Endocytosis | ko04144 | K03283 | heat shock 70kDa protein 1/8 |
| DN77142c2_g1 | Cellular Processes | Transport and catabolism | Endocytosis | ko04144 | K03283 | heat shock 70kDa protein 1/8 |
| DN57418c0_g4 | Cellular Processes | Transport and catabolism | Endocytosis | ko04144 | K03283 | heat shock 70kDa protein 1/8 |
| DN93137c3_g3 | Cellular Processes | Transport and catabolism | Endocytosis | ko04144 | K03283 | heat shock 70kDa protein 1/8 |
| DN93137c1_g1 | Cellular Processes | Transport and catabolism | Endocytosis | ko04144 | K03283 | heat shock 70kDa protein 1/8 |
| DN93947c5_g1 | Cellular Processes | Transport and catabolism | Endocytosis | ko04144 | K03283 | heat shock 70kDa protein 1/8 |
| DN68039c1_g1 | Cellular Processes | Transport and catabolism | Endocytosis | ko04144 | K03283 | heat shock 70kDa protein 1/8 |
| DN62210c0_g1 | Cellular Processes | Transport and catabolism | Endocytosis | ko04144 | K03283 | heat shock 70kDa protein 1/8 |
| DN93137c2_g2 | Cellular Processes | Transport and catabolism | Endocytosis | ko04144 | K03283 | heat shock 70kDa protein 1/8 |
| DN56932c1_g1 | Cellular Processes | Transport and catabolism | Endocytosis | ko04144 | K03283 | heat shock 70kDa protein 1/8 |
| DN60318c1_g1 | Cellular Processes | Transport and catabolism | Endocytosis | ko04144 | K03283 | heat shock 70kDa protein 1/8 |
| DN72847c1_g1 | Cellular Processes | Transport and catabolism | Endocytosis | ko04144 | K03283 | heat shock 70kDa protein 1/8 |
| DN67597c2_g3 | Cellular Processes | Transport and catabolism | Endocytosis | ko04144 | K03283 | heat shock 70kDa protein 1/8 |
| DN81155c1_g2 | Cellular Processes | Transport and catabolism | Endocytosis | ko04144 | K03283 | heat shock 70kDa protein 1/8 |
| DN81155c1_g3 | Cellular Processes | Transport and catabolism | Endocytosis | ko04144 | K03283 | heat shock 70kDa protein 1/8 |
| DN61177c2_g1 | Cellular Processes | Transport and catabolism | Endocytosis | ko04144 | K10365 | capping protein (actin filament) muscle Z-line, beta |
| DN73947c0_g6 | Cellular Processes | Transport and catabolism | Phagosome | ko04145 | K02153 | V-type H+-transporting ATPase subunit e |
| DN89461c0_g3 | Cellular Processes | Transport and catabolism | Peroxisome | ko04146 | K04564 | superoxide dismutase, Fe-Mn family |
| DN89461c0_g2 | Cellular Processes | Transport and catabolism | Peroxisome | ko04146 | K04564 | superoxide dismutase, Fe-Mn family |
| DN84451c0_g2 | Environmental Information Processing | Signal transduction | Plant hormone signal transduction | ko04075 | K14432 | ABA responsive element binding factor |
| DN83606c0_g2 | Environmental Information Processing | Signal transduction | Plant hormone signal transduction | ko04075 | K14432 | ABA responsive element binding factor |
| DN91097c1_g3 | Environmental Information Processing | Signal transduction | Plant hormone signal transduction | ko04075 | K14510 | serine/threonine-protein kinase CTR1 |
| DN96191c2_g1 | Environmental Information Processing | Signal transduction | Plant hormone signal transduction | ko04075 | K14510 | serine/threonine-protein kinase CTR1 |
| DN77549c1_g2 | Environmental Information Processing | Signal transduction | Plant hormone signal transduction | ko04075 | K00737 | beta-1,4-mannosyl-glycoprotein beta-1,4-N-acetylglucosaminyltransferase |
| DN84171c0_g1 | Genetic Information Processing | Folding, sorting and degradation | RNA degradation | ko03018 | K04043 | molecular chaperone DnaK |
| DN93153c0_g3 | Genetic Information Processing | Folding, sorting and degradation | RNA degradation | ko03018 | K04043 | molecular chaperone DnaK |
| DN89495c1_g2 | Genetic Information Processing | Folding, sorting and degradation | RNA degradation | ko03018 | K04077 | chaperonin GroEL |
| DN81599c6_g1 | Genetic Information Processing | Folding, sorting and degradation | RNA degradation | ko03018 | K04077 | chaperonin GroEL |
| DN88206c0_g1 | Genetic Information Processing | Folding, sorting and degradation | RNA degradation | ko03018 | K04077 | chaperonin GroEL |
| DN77822c0_g1 | Genetic Information Processing | Folding, sorting and degradation | RNA degradation | ko03018 | K04077 | chaperonin GroEL |
| DN94362c3_g1 | Genetic Information Processing | Folding, sorting and degradation | RNA degradation | ko03018 | K04077 | chaperonin GroEL |
| DN79352c2_g1 | Genetic Information Processing | Folding, sorting and degradation | RNA degradation | ko03018 | K04077 | chaperonin GroEL |
| DN78098c0_g1 | Genetic Information Processing | Folding, sorting and degradation | RNA degradation | ko03018 | K04077 | chaperonin GroEL |
| DN57472c0_g1 | Genetic Information Processing | Folding, sorting and degradation | Ubiquitin mediated proteolysis | ko04120 | K03094 | S-phase kinase-associated protein 1 |
| DN91964c0_g1 | Genetic Information Processing | Folding, sorting and degradation | Ubiquitin mediated proteolysis | ko04120 | K03363 | cell division cycle 20, cofactor of APC complex |
| DN96650c10_g2 | Genetic Information Processing | Folding, sorting and degradation | Ubiquitin mediated proteolysis | ko04120 | K10144 | RING finger and CHY zinc finger domain-containing protein 1 |
| DN57472c0_g1 | Genetic Information Processing | Folding, sorting and degradation | Protein processing in endoplasmic reticulum | ko04141 | K03094 | S-phase kinase-associated protein 1 |
| DN63694c0_g3 | Genetic Information Processing | Folding, sorting and degradation | Protein processing in endoplasmic reticulum | ko04141 | K03283 | heat shock 70kDa protein 1/8 |
| DN72862c2_g3 | Genetic Information Processing | Folding, sorting and degradation | Protein processing in endoplasmic reticulum | ko04141 | K03283 | heat shock 70kDa protein 1/8 |
| DN58604c2_g2 | Genetic Information Processing | Folding, sorting and degradation | Protein processing in endoplasmic reticulum | ko04141 | K03283 | heat shock 70kDa protein 1/8 |
| DN63694c0_g2 | Genetic Information Processing | Folding, sorting and degradation | Protein processing in endoplasmic reticulum | ko04141 | K03283 | heat shock 70kDa protein 1/8 |
| DN77812c1_g1 | Genetic Information Processing | Folding, sorting and degradation | Protein processing in endoplasmic reticulum | ko04141 | K03283 | heat shock 70kDa protein 1/8 |
| DN72862c2_g1 | Genetic Information Processing | Folding, sorting and degradation | Protein processing in endoplasmic reticulum | ko04141 | K03283 | heat shock 70kDa protein 1/8 |
| DN70478c3_g1 | Genetic Information Processing | Folding, sorting and degradation | Protein processing in endoplasmic reticulum | ko04141 | K03283 | heat shock 70kDa protein 1/8 |
| DN63724c1_g1 | Genetic Information Processing | Folding, sorting and degradation | Protein processing in endoplasmic reticulum | ko04141 | K03283 | heat shock 70kDa protein 1/8 |
| DN63694c0_g1 | Genetic Information Processing | Folding, sorting and degradation | Protein processing in endoplasmic reticulum | ko04141 | K03283 | heat shock 70kDa protein 1/8 |
| DN76620c0_g2 | Genetic Information Processing | Folding, sorting and degradation | Protein processing in endoplasmic reticulum | ko04141 | K03283 | heat shock 70kDa protein 1/8 |
| DN77142c1_g1 | Genetic Information Processing | Folding, sorting and degradation | Protein processing in endoplasmic reticulum | ko04141 | K03283 | heat shock 70kDa protein 1/8 |
| DN70992c0_g1 | Genetic Information Processing | Folding, sorting and degradation | Protein processing in endoplasmic reticulum | ko04141 | K03283 | heat shock 70kDa protein 1/8 |
| DN77142c2_g2 | Genetic Information Processing | Folding, sorting and degradation | Protein processing in endoplasmic reticulum | ko04141 | K03283 | heat shock 70kDa protein 1/8 |
| DN81155c1_g6 | Genetic Information Processing | Folding, sorting and degradation | Protein processing in endoplasmic reticulum | ko04141 | K03283 | heat shock 70kDa protein 1/8 |
| DN56797c0_g1 | Genetic Information Processing | Folding, sorting and degradation | Protein processing in endoplasmic reticulum | ko04141 | K03283 | heat shock 70kDa protein 1/8 |
| DN65094c0_g1 | Genetic Information Processing | Folding, sorting and degradation | Protein processing in endoplasmic reticulum | ko04141 | K03283 | heat shock 70kDa protein 1/8 |
| DN95710c3_g2 | Genetic Information Processing | Folding, sorting and degradation | Protein processing in endoplasmic reticulum | ko04141 | K03283 | heat shock 70kDa protein 1/8 |
| DN77142c2_g1 | Genetic Information Processing | Folding, sorting and degradation | Protein processing in endoplasmic reticulum | ko04141 | K03283 | heat shock 70kDa protein 1/8 |
| DN57418c0_g4 | Genetic Information Processing | Folding, sorting and degradation | Protein processing in endoplasmic reticulum | ko04141 | K03283 | heat shock 70kDa protein 1/8 |
| DN93137c3_g3 | Genetic Information Processing | Folding, sorting and degradation | Protein processing in endoplasmic reticulum | ko04141 | K03283 | heat shock 70kDa protein 1/8 |
| DN93137c1_g1 | Genetic Information Processing | Folding, sorting and degradation | Protein processing in endoplasmic reticulum | ko04141 | K03283 | heat shock 70kDa protein 1/8 |
| DN93947c5_g1 | Genetic Information Processing | Folding, sorting and degradation | Protein processing in endoplasmic reticulum | ko04141 | K03283 | heat shock 70kDa protein 1/8 |
| DN68039c1_g1 | Genetic Information Processing | Folding, sorting and degradation | Protein processing in endoplasmic reticulum | ko04141 | K03283 | heat shock 70kDa protein 1/8 |
| DN62210c0_g1 | Genetic Information Processing | Folding, sorting and degradation | Protein processing in endoplasmic reticulum | ko04141 | K03283 | heat shock 70kDa protein 1/8 |
| DN93137c2_g2 | Genetic Information Processing | Folding, sorting and degradation | Protein processing in endoplasmic reticulum | ko04141 | K03283 | heat shock 70kDa protein 1/8 |
| DN56932c1_g1 | Genetic Information Processing | Folding, sorting and degradation | Protein processing in endoplasmic reticulum | ko04141 | K03283 | heat shock 70kDa protein 1/8 |
| DN60318c1_g1 | Genetic Information Processing | Folding, sorting and degradation | Protein processing in endoplasmic reticulum | ko04141 | K03283 | heat shock 70kDa protein 1/8 |
| DN72847c1_g1 | Genetic Information Processing | Folding, sorting and degradation | Protein processing in endoplasmic reticulum | ko04141 | K03283 | heat shock 70kDa protein 1/8 |
| DN67597c2_g3 | Genetic Information Processing | Folding, sorting and degradation | Protein processing in endoplasmic reticulum | ko04141 | K03283 | heat shock 70kDa protein 1/8 |
| DN81155c1_g2 | Genetic Information Processing | Folding, sorting and degradation | Protein processing in endoplasmic reticulum | ko04141 | K03283 | heat shock 70kDa protein 1/8 |
| DN81155c1_g3 | Genetic Information Processing | Folding, sorting and degradation | Protein processing in endoplasmic reticulum | ko04141 | K03283 | heat shock 70kDa protein 1/8 |
| DN61807c0_g1 | Genetic Information Processing | Folding, sorting and degradation | Protein processing in endoplasmic reticulum | ko04141 | K04079 | molecular chaperone HtpG |
| DN75960c2_g1 | Genetic Information Processing | Folding, sorting and degradation | Protein processing in endoplasmic reticulum | ko04141 | K04079 | molecular chaperone HtpG |
| DN65124c0_g3 | Genetic Information Processing | Folding, sorting and degradation | Protein processing in endoplasmic reticulum | ko04141 | K04079 | molecular chaperone HtpG |
| DN57598c1_g1 | Genetic Information Processing | Folding, sorting and degradation | Protein processing in endoplasmic reticulum | ko04141 | K04079 | molecular chaperone HtpG |
| DN70663c1_g1 | Genetic Information Processing | Folding, sorting and degradation | Protein processing in endoplasmic reticulum | ko04141 | K04079 | molecular chaperone HtpG |
| DN94152c0_g3 | Genetic Information Processing | Folding, sorting and degradation | Protein processing in endoplasmic reticulum | ko04141 | K04079 | molecular chaperone HtpG |
| DN61807c1_g1 | Genetic Information Processing | Folding, sorting and degradation | Protein processing in endoplasmic reticulum | ko04141 | K04079 | molecular chaperone HtpG |
| DN60687c0_g2 | Genetic Information Processing | Folding, sorting and degradation | Protein processing in endoplasmic reticulum | ko04141 | K04079 | molecular chaperone HtpG |
| DN95029c3_g1 | Genetic Information Processing | Folding, sorting and degradation | Protein processing in endoplasmic reticulum | ko04141 | K04079 | molecular chaperone HtpG |
| DN70663c2_g1 | Genetic Information Processing | Folding, sorting and degradation | Protein processing in endoplasmic reticulum | ko04141 | K04079 | molecular chaperone HtpG |
| DN96189c5_g1 | Genetic Information Processing | Folding, sorting and degradation | Protein processing in endoplasmic reticulum | ko04141 | K04079 | molecular chaperone HtpG |
| DN95732c2_g2 | Genetic Information Processing | Folding, sorting and degradation | Protein processing in endoplasmic reticulum | ko04141 | K04079 | molecular chaperone HtpG |
| DN65611c1_g3 | Genetic Information Processing | Folding, sorting and degradation | Protein processing in endoplasmic reticulum | ko04141 | K04079 | molecular chaperone HtpG |
| DN65478c2_g3 | Genetic Information Processing | Folding, sorting and degradation | Protein processing in endoplasmic reticulum | ko04141 | K04079 | molecular chaperone HtpG |
| DN93355c3_g1 | Genetic Information Processing | Folding, sorting and degradation | Protein processing in endoplasmic reticulum | ko04141 | K04079 | molecular chaperone HtpG |
| DN65611c1_g1 | Genetic Information Processing | Folding, sorting and degradation | Protein processing in endoplasmic reticulum | ko04141 | K04079 | molecular chaperone HtpG |
| DN65968c1_g1 | Genetic Information Processing | Folding, sorting and degradation | Protein processing in endoplasmic reticulum | ko04141 | K04079 | molecular chaperone HtpG |
| DN94152c0_g4 | Genetic Information Processing | Folding, sorting and degradation | Protein processing in endoplasmic reticulum | ko04141 | K04079 | molecular chaperone HtpG |
| DN94152c1_g1 | Genetic Information Processing | Folding, sorting and degradation | Protein processing in endoplasmic reticulum | ko04141 | K04079 | molecular chaperone HtpG |
| DN95029c3_g3 | Genetic Information Processing | Folding, sorting and degradation | Protein processing in endoplasmic reticulum | ko04141 | K04079 | molecular chaperone HtpG |
| DN95640c5_g2 | Genetic Information Processing | Folding, sorting and degradation | Protein processing in endoplasmic reticulum | ko04141 | K09487 | heat shock protein 90kDa beta |
| DN95640c5_g1 | Genetic Information Processing | Folding, sorting and degradation | Protein processing in endoplasmic reticulum | ko04141 | K09487 | heat shock protein 90kDa beta |
| DN57042c2_g1 | Genetic Information Processing | Folding, sorting and degradation | Protein processing in endoplasmic reticulum | ko04141 | K09503 | DnaJ homolog subfamily A member 2 |
| DN63915c1_g1 | Genetic Information Processing | Folding, sorting and degradation | Protein processing in endoplasmic reticulum | ko04141 | K09503 | DnaJ homolog subfamily A member 2 |
| DN86433c2_g1 | Genetic Information Processing | Folding, sorting and degradation | Protein processing in endoplasmic reticulum | ko04141 | K09562 | hsp70-interacting protein |
| DN92750c4_g4 | Genetic Information Processing | Folding, sorting and degradation | Protein processing in endoplasmic reticulum | ko04141 | K13525 | transitional endoplasmic reticulum ATPase |
| DN93060c4_g3 | Genetic Information Processing | Folding, sorting and degradation | Protein processing in endoplasmic reticulum | ko04141 | K13525 | transitional endoplasmic reticulum ATPase |
| DN62302c2_g1 | Genetic Information Processing | Folding, sorting and degradation | Protein processing in endoplasmic reticulum | ko04141 | K13525 | transitional endoplasmic reticulum ATPase |
| DN92750c3_g1 | Genetic Information Processing | Folding, sorting and degradation | Protein processing in endoplasmic reticulum | ko04141 | K13525 | transitional endoplasmic reticulum ATPase |
| DN92750c4_g2 | Genetic Information Processing | Folding, sorting and degradation | Protein processing in endoplasmic reticulum | ko04141 | K13525 | transitional endoplasmic reticulum ATPase |
| DN58389c0_g1 | Genetic Information Processing | Folding, sorting and degradation | Protein processing in endoplasmic reticulum | ko04141 | K13525 | transitional endoplasmic reticulum ATPase |
| DN61038c2_g3 | Genetic Information Processing | Folding, sorting and degradation | Protein processing in endoplasmic reticulum | ko04141 | K13525 | transitional endoplasmic reticulum ATPase |
| DN61038c2_g1 | Genetic Information Processing | Folding, sorting and degradation | Protein processing in endoplasmic reticulum | ko04141 | K13525 | transitional endoplasmic reticulum ATPase |
| DN96290c1_g1 | Genetic Information Processing | Folding, sorting and degradation | Protein processing in endoplasmic reticulum | ko04141 | K13525 | transitional endoplasmic reticulum ATPase |
| DN73249c2_g2 | Genetic Information Processing | Folding, sorting and degradation | Protein processing in endoplasmic reticulum | ko04141 | K13525 | transitional endoplasmic reticulum ATPase |
| DN86693c1_g1 | Genetic Information Processing | Folding, sorting and degradation | Protein processing in endoplasmic reticulum | ko04141 | K13525 | transitional endoplasmic reticulum ATPase |
| DN87137c4_g2 | Genetic Information Processing | Folding, sorting and degradation | Protein processing in endoplasmic reticulum | ko04141 | K13525 | transitional endoplasmic reticulum ATPase |
| DN96290c2_g2 | Genetic Information Processing | Folding, sorting and degradation | Protein processing in endoplasmic reticulum | ko04141 | K13525 | transitional endoplasmic reticulum ATPase |
| DN78461c0_g2 | Genetic Information Processing | Folding, sorting and degradation | Protein processing in endoplasmic reticulum | ko04141 | K13525 | transitional endoplasmic reticulum ATPase |
| DN80405c0_g1 | Genetic Information Processing | Folding, sorting and degradation | Protein processing in endoplasmic reticulum | ko04141 | K13525 | transitional endoplasmic reticulum ATPase |
| DN92970c2_g3 | Genetic Information Processing | Folding, sorting and degradation | Protein processing in endoplasmic reticulum | ko04141 | K13989 | Derlin-2/3 |
| DN59349c0_g1 | Genetic Information Processing | Folding, sorting and degradation | Protein processing in endoplasmic reticulum | ko04141 | K13993 | HSP20 family protein |
| DN64934c0_g1 | Genetic Information Processing | Folding, sorting and degradation | Protein processing in endoplasmic reticulum | ko04141 | K13993 | HSP20 family protein |
| DN76453c2_g1 | Genetic Information Processing | Transcription | RNA polymerase | ko03020 | K03006 | DNA-directed RNA polymerase II subunit RPB1 |
| DN63694c0_g3 | Genetic Information Processing | Transcription | Spliceosome | ko03040 | K03283 | heat shock 70kDa protein 1/8 |
| DN72862c2_g3 | Genetic Information Processing | Transcription | Spliceosome | ko03040 | K03283 | heat shock 70kDa protein 1/8 |
| DN58604c2_g2 | Genetic Information Processing | Transcription | Spliceosome | ko03040 | K03283 | heat shock 70kDa protein 1/8 |
| DN63694c0_g2 | Genetic Information Processing | Transcription | Spliceosome | ko03040 | K03283 | heat shock 70kDa protein 1/8 |
| DN77812c1_g1 | Genetic Information Processing | Transcription | Spliceosome | ko03040 | K03283 | heat shock 70kDa protein 1/8 |
| DN72862c2_g1 | Genetic Information Processing | Transcription | Spliceosome | ko03040 | K03283 | heat shock 70kDa protein 1/8 |
| DN70478c3_g1 | Genetic Information Processing | Transcription | Spliceosome | ko03040 | K03283 | heat shock 70kDa protein 1/8 |
| DN63724c1_g1 | Genetic Information Processing | Transcription | Spliceosome | ko03040 | K03283 | heat shock 70kDa protein 1/8 |
| DN63694c0_g1 | Genetic Information Processing | Transcription | Spliceosome | ko03040 | K03283 | heat shock 70kDa protein 1/8 |
| DN76620c0_g2 | Genetic Information Processing | Transcription | Spliceosome | ko03040 | K03283 | heat shock 70kDa protein 1/8 |
| DN77142c1_g1 | Genetic Information Processing | Transcription | Spliceosome | ko03040 | K03283 | heat shock 70kDa protein 1/8 |
| DN70992c0_g1 | Genetic Information Processing | Transcription | Spliceosome | ko03040 | K03283 | heat shock 70kDa protein 1/8 |
| DN77142c2_g2 | Genetic Information Processing | Transcription | Spliceosome | ko03040 | K03283 | heat shock 70kDa protein 1/8 |
| DN81155c1_g6 | Genetic Information Processing | Transcription | Spliceosome | ko03040 | K03283 | heat shock 70kDa protein 1/8 |
| DN56797c0_g1 | Genetic Information Processing | Transcription | Spliceosome | ko03040 | K03283 | heat shock 70kDa protein 1/8 |
| DN65094c0_g1 | Genetic Information Processing | Transcription | Spliceosome | ko03040 | K03283 | heat shock 70kDa protein 1/8 |
| DN95710c3_g2 | Genetic Information Processing | Transcription | Spliceosome | ko03040 | K03283 | heat shock 70kDa protein 1/8 |
| DN77142c2_g1 | Genetic Information Processing | Transcription | Spliceosome | ko03040 | K03283 | heat shock 70kDa protein 1/8 |
| DN57418c0_g4 | Genetic Information Processing | Transcription | Spliceosome | ko03040 | K03283 | heat shock 70kDa protein 1/8 |
| DN93137c3_g3 | Genetic Information Processing | Transcription | Spliceosome | ko03040 | K03283 | heat shock 70kDa protein 1/8 |
| DN93137c1_g1 | Genetic Information Processing | Transcription | Spliceosome | ko03040 | K03283 | heat shock 70kDa protein 1/8 |
| DN93947c5_g1 | Genetic Information Processing | Transcription | Spliceosome | ko03040 | K03283 | heat shock 70kDa protein 1/8 |
| DN68039c1_g1 | Genetic Information Processing | Transcription | Spliceosome | ko03040 | K03283 | heat shock 70kDa protein 1/8 |
| DN62210c0_g1 | Genetic Information Processing | Transcription | Spliceosome | ko03040 | K03283 | heat shock 70kDa protein 1/8 |
| DN93137c2_g2 | Genetic Information Processing | Transcription | Spliceosome | ko03040 | K03283 | heat shock 70kDa protein 1/8 |
| DN56932c1_g1 | Genetic Information Processing | Transcription | Spliceosome | ko03040 | K03283 | heat shock 70kDa protein 1/8 |
| DN60318c1_g1 | Genetic Information Processing | Transcription | Spliceosome | ko03040 | K03283 | heat shock 70kDa protein 1/8 |
| DN72847c1_g1 | Genetic Information Processing | Transcription | Spliceosome | ko03040 | K03283 | heat shock 70kDa protein 1/8 |
| DN67597c2_g3 | Genetic Information Processing | Transcription | Spliceosome | ko03040 | K03283 | heat shock 70kDa protein 1/8 |
| DN81155c1_g2 | Genetic Information Processing | Transcription | Spliceosome | ko03040 | K03283 | heat shock 70kDa protein 1/8 |
| DN81155c1_g3 | Genetic Information Processing | Transcription | Spliceosome | ko03040 | K03283 | heat shock 70kDa protein 1/8 |
| DN85353c3_g1 | Genetic Information Processing | Transcription | Spliceosome | ko03040 | K12836 | splicing factor U2AF 35 kDa subunit |
| DN93999c1_g3 | Genetic Information Processing | Transcription | Spliceosome | ko03040 | K12897 | transformer-2 protein |
| DN59558c0_g1 | Genetic Information Processing | Translation | Ribosome | ko03010 | K02977 | small subunit ribosomal protein S27Ae |
| DN90233c1_g1 | Genetic Information Processing | Translation | RNA transport | ko03013 | K14298 | mRNA export factor |
| DN86926c1_g1 | Genetic Information Processing | Translation | mRNA surveillance pathway | ko03015 | K14376 | poly(A) polymerase |
| DN89401c0_g1 | Metabolism | Amino acid metabolism | Glycine, serine and threonine metabolism | ko00260 | K01696 | tryptophan synthase beta chain |
| DN90773c2_g1 | Metabolism | Amino acid metabolism | Glycine, serine and threonine metabolism | ko00260 | K12524 | bifunctional aspartokinase / homoserine dehydrogenase 1 |
| DN90773c2_g1 | Metabolism | Amino acid metabolism | Cysteine and methionine metabolism | ko00270 | K12524 | bifunctional aspartokinase / homoserine dehydrogenase 1 |
| DN72271c4_g2 | Metabolism | Amino acid metabolism | Valine, leucine and isoleucine degradation | ko00280 | K05605 | 3-hydroxyisobutyryl-CoA hydrolase |
| DN90773c2_g1 | Metabolism | Amino acid metabolism | Lysine biosynthesis | ko00300 | K12524 | bifunctional aspartokinase / homoserine dehydrogenase 1 |
| DN89401c0_g1 | Metabolism | Amino acid metabolism | Phenylalanine, tyrosine and tryptophan biosynthesis | ko00400 | K01696 | tryptophan synthase beta chain |
| DN90773c2_g1 | Metabolism | Biosynthesis of other secondary metabolites | Monobactam biosynthesis | ko00261 | K12524 | bifunctional aspartokinase / homoserine dehydrogenase 1 |
| DN95273c1_g2 | Metabolism | Carbohydrate metabolism | Amino sugar and nucleotide sugar metabolism | ko00520 | K00326 | cytochrome-b5 reductase |
| DN72271c4_g2 | Metabolism | Carbohydrate metabolism | Propanoate metabolism | ko00640 | K05605 | 3-hydroxyisobutyryl-CoA hydrolase |
| DN73947c0_g6 | Metabolism | Energy metabolism | Oxidative phosphorylation | ko00190 | K02153 | V-type H+-transporting ATPase subunit e |
| DN81377c0_g1 | Metabolism | Energy metabolism | Oxidative phosphorylation | ko00190 | K03883 | NADH-ubiquinone oxidoreductase chain 5 |
| DN57936c0_g1 | Metabolism | Energy metabolism | Oxidative phosphorylation | ko00190 | K03940 | NADH dehydrogenase (ubiquinone) Fe-S protein 7 |
| DN89549c1_g2 | Metabolism | Energy metabolism | Oxidative phosphorylation | ko00190 | K03942 | NADH dehydrogenase (ubiquinone) flavoprotein 1 |
| DN91247c0_g1 | Metabolism | Energy metabolism | Photosynthesis | ko00195 | K02690 | photosystem I P700 chlorophyll a apoprotein A2 |
| DN77549c1_g2 | Metabolism | Glycan biosynthesis and metabolism | N-Glycan biosynthesis | ko00510 | K00737 | beta-1,4-mannosyl-glycoprotein beta-1,4-N-acetylglucosaminyltransferase |
| DN93033c1_g2 | Metabolism | Lipid metabolism | Glycerophospholipid metabolism | ko00564 | K06130 | lysophospholipase II |
| DN62247c0_g1 | Metabolism | Lipid metabolism | Arachidonic acid metabolism | ko00590 | K15730 | cytosolic prostaglandin-E synthase |
| DN93263c1_g1 | Metabolism | Lipid metabolism | Arachidonic acid metabolism | ko00590 | K15730 | cytosolic prostaglandin-E synthase |
| DN84229c1_g4 | Metabolism | Metabolism of cofactors and vitamins | Ubiquinone and other terpenoid-quinone biosynthesis | ko00130 | K03809 | NAD(P)H dehydrogenase (quinone) |
| DN86356c0_g2 | Metabolism | Metabolism of cofactors and vitamins | Vitamin B6 metabolism | ko00750 | K06215 | pyridoxal 5'-phosphate synthase pdxS subunit |
| DN86356c0_g1 | Metabolism | Metabolism of cofactors and vitamins | Vitamin B6 metabolism | ko00750 | K06215 | pyridoxal 5'-phosphate synthase pdxS subunit |
| DN86356c0_g3 | Metabolism | Metabolism of cofactors and vitamins | Vitamin B6 metabolism | ko00750 | K06215 | pyridoxal 5'-phosphate synthase pdxS subunit |
| DN74989c2_g2 | Metabolism | Metabolism of cofactors and vitamins | Nicotinate and nicotinamide metabolism | ko00760 | K00763 | nicotinate phosphoribosyltransferase |
| DN74602c0_g2 | Metabolism | Metabolism of cofactors and vitamins | Pantothenate and CoA biosynthesis | ko00770 | K09680 | type II pantothenate kinase |
| DN72271c4_g2 | Metabolism | Metabolism of other amino acids | beta-Alanine metabolism | ko00410 | K05605 | 3-hydroxyisobutyryl-CoA hydrolase |
| DN94108c3_g1 | Metabolism | Nucleotide metabolism | Purine metabolism | ko00230 | K01514 | exopolyphosphatase |
| DN76453c2_g1 | Metabolism | Nucleotide metabolism | Purine metabolism | ko00230 | K03006 | DNA-directed RNA polymerase II subunit RPB1 |
| DN76453c2_g1 | Metabolism | Nucleotide metabolism | Pyrimidine metabolism | ko00240 | K03006 | DNA-directed RNA polymerase II subunit RPB1 |
| DN72271c4_g2 | Metabolism | Overview | Carbon metabolism | ko01200 | K05605 | 3-hydroxyisobutyryl-CoA hydrolase |
| DN89401c0_g1 | Metabolism | Overview | Biosynthesis of amino acids | ko01230 | K01696 | tryptophan synthase beta chain |
| DN90773c2_g1 | Metabolism | Overview | Biosynthesis of amino acids | ko01230 | K12524 | bifunctional aspartokinase / homoserine dehydrogenase 1 |
| DN61807c0_g1 | Organismal Systems | Environmental adaptation | Plant-pathogen interaction | ko04626 | K04079 | molecular chaperone HtpG |
| DN75960c2_g1 | Organismal Systems | Environmental adaptation | Plant-pathogen interaction | ko04626 | K04079 | molecular chaperone HtpG |
| DN65124c0_g3 | Organismal Systems | Environmental adaptation | Plant-pathogen interaction | ko04626 | K04079 | molecular chaperone HtpG |
| DN57598c1_g1 | Organismal Systems | Environmental adaptation | Plant-pathogen interaction | ko04626 | K04079 | molecular chaperone HtpG |
| DN70663c1_g1 | Organismal Systems | Environmental adaptation | Plant-pathogen interaction | ko04626 | K04079 | molecular chaperone HtpG |
| DN94152c0_g3 | Organismal Systems | Environmental adaptation | Plant-pathogen interaction | ko04626 | K04079 | molecular chaperone HtpG |
| DN61807c1_g1 | Organismal Systems | Environmental adaptation | Plant-pathogen interaction | ko04626 | K04079 | molecular chaperone HtpG |
| DN60687c0_g2 | Organismal Systems | Environmental adaptation | Plant-pathogen interaction | ko04626 | K04079 | molecular chaperone HtpG |
| DN95029c3_g1 | Organismal Systems | Environmental adaptation | Plant-pathogen interaction | ko04626 | K04079 | molecular chaperone HtpG |
| DN70663c2_g1 | Organismal Systems | Environmental adaptation | Plant-pathogen interaction | ko04626 | K04079 | molecular chaperone HtpG |
| DN96189c5_g1 | Organismal Systems | Environmental adaptation | Plant-pathogen interaction | ko04626 | K04079 | molecular chaperone HtpG |
| DN95732c2_g2 | Organismal Systems | Environmental adaptation | Plant-pathogen interaction | ko04626 | K04079 | molecular chaperone HtpG |
| DN65611c1_g3 | Organismal Systems | Environmental adaptation | Plant-pathogen interaction | ko04626 | K04079 | molecular chaperone HtpG |
| DN65478c2_g3 | Organismal Systems | Environmental adaptation | Plant-pathogen interaction | ko04626 | K04079 | molecular chaperone HtpG |
| DN93355c3_g1 | Organismal Systems | Environmental adaptation | Plant-pathogen interaction | ko04626 | K04079 | molecular chaperone HtpG |
| DN65611c1_g1 | Organismal Systems | Environmental adaptation | Plant-pathogen interaction | ko04626 | K04079 | molecular chaperone HtpG |
| DN65968c1_g1 | Organismal Systems | Environmental adaptation | Plant-pathogen interaction | ko04626 | K04079 | molecular chaperone HtpG |
| DN94152c0_g4 | Organismal Systems | Environmental adaptation | Plant-pathogen interaction | ko04626 | K04079 | molecular chaperone HtpG |
| DN94152c1_g1 | Organismal Systems | Environmental adaptation | Plant-pathogen interaction | ko04626 | K04079 | molecular chaperone HtpG |
| DN95029c3_g3 | Organismal Systems | Environmental adaptation | Plant-pathogen interaction | ko04626 | K04079 | molecular chaperone HtpG |
| DN95640c5_g2 | Organismal Systems | Environmental adaptation | Plant-pathogen interaction | ko04626 | K09487 | heat shock protein 90kDa beta |
| DN95640c5_g1 | Organismal Systems | Environmental adaptation | Plant-pathogen interaction | ko04626 | K09487 | heat shock protein 90kDa beta |

**Table S4 KEGG annotation of genes in the black module**

| **Gene ID** | **1st-level (pathway)** | **2nd-level (pathway)** | **3rd-level (pathway)** | **Pathway id** | **KO id** | **KO Description** |
| --- | --- | --- | --- | --- | --- | --- |
| DN76620c0_g1 | Cellular Processes | Transport and catabolism | Endocytosis | ko04144 | K03283 | heat shock 70kDa protein 1/8 |
| DN77812c0_g1 | Cellular Processes | Transport and catabolism | Endocytosis | ko04144 | K03283 | heat shock 70kDa protein 1/8 |
| DN93137c2_g1 | Cellular Processes | Transport and catabolism | Endocytosis | ko04144 | K03283 | heat shock 70kDa protein 1/8 |
| DN58952c0_g5 | Cellular Processes | Transport and catabolism | Endocytosis | ko04144 | K03283 | heat shock 70kDa protein 1/8 |
| DN84857c0_g2 | Cellular Processes | Transport and catabolism | Endocytosis | ko04144 | K03283 | heat shock 70kDa protein 1/8 |
| DN56932c1_g2 | Cellular Processes | Transport and catabolism | Endocytosis | ko04144 | K03283 | heat shock 70kDa protein 1/8 |
| DN83606c0_g4 | Environmental Information Processing | Signal transduction | Plant hormone signal transduction | ko04075 | K14432 | ABA responsive element binding factor |
| DN95810c2_g1 | Environmental Information Processing | Signal transduction | Plant hormone signal transduction | ko04075 | K14485 | transport inhibitor response 1 |
| DN80122c0_g2 | Genetic Information Processing | Folding, sorting and degradation | RNA degradation | ko03018 | K04077 | chaperonin GroEL |
| DN64152c2_g1 | Genetic Information Processing | Folding, sorting and degradation | RNA degradation | ko03018 | K04077 | chaperonin GroEL |
| DN64152c1_g1 | Genetic Information Processing | Folding, sorting and degradation | RNA degradation | ko03018 | K04077 | chaperonin GroEL |
| DN79352c3_g3 | Genetic Information Processing | Folding, sorting and degradation | RNA degradation | ko03018 | K04077 | chaperonin GroEL |
| DN95776c3_g8 | Genetic Information Processing | Folding, sorting and degradation | RNA degradation | ko03018 | K04077 | chaperonin GroEL |
| DN74637c3_g1 | Genetic Information Processing | Folding, sorting and degradation | RNA degradation | ko03018 | K04077 | chaperonin GroEL |
| DN95607c2_g6 | Genetic Information Processing | Folding, sorting and degradation | RNA degradation | ko03018 | K04077 | chaperonin GroEL |
| DN87907c0_g3 | Genetic Information Processing | Folding, sorting and degradation | Proteasome | ko03050 | K03037 | 26S proteasome regulatory subunit N7 |
| DN64917c1_g1 | Genetic Information Processing | Folding, sorting and degradation | Proteasome | ko03050 | K03062 | 26S proteasome regulatory subunit T2 |
| DN81535c3_g2 | Genetic Information Processing | Folding, sorting and degradation | Ubiquitin mediated proteolysis | ko04120 | K03868 | RING-box protein 1 |
| DN96482c4_g1 | Genetic Information Processing | Folding, sorting and degradation | SNARE interactions in vesicular transport | ko04130 | K08503 | syntaxin of plants SYP5 |
| DN76620c0_g1 | Genetic Information Processing | Folding, sorting and degradation | Protein processing in endoplasmic reticulum | ko04141 | K03283 | heat shock 70kDa protein 1/8 |
| DN77812c0_g1 | Genetic Information Processing | Folding, sorting and degradation | Protein processing in endoplasmic reticulum | ko04141 | K03283 | heat shock 70kDa protein 1/8 |
| DN93137c2_g1 | Genetic Information Processing | Folding, sorting and degradation | Protein processing in endoplasmic reticulum | ko04141 | K03283 | heat shock 70kDa protein 1/8 |
| DN58952c0_g5 | Genetic Information Processing | Folding, sorting and degradation | Protein processing in endoplasmic reticulum | ko04141 | K03283 | heat shock 70kDa protein 1/8 |
| DN84857c0_g2 | Genetic Information Processing | Folding, sorting and degradation | Protein processing in endoplasmic reticulum | ko04141 | K03283 | heat shock 70kDa protein 1/8 |
| DN56932c1_g2 | Genetic Information Processing | Folding, sorting and degradation | Protein processing in endoplasmic reticulum | ko04141 | K03283 | heat shock 70kDa protein 1/8 |
| DN81535c3_g2 | Genetic Information Processing | Folding, sorting and degradation | Protein processing in endoplasmic reticulum | ko04141 | K03868 | RING-box protein 1 |
| DN84227c0_g3 | Genetic Information Processing | Folding, sorting and degradation | Protein processing in endoplasmic reticulum | ko04141 | K09487 | heat shock protein 90kDa beta |
| DN76833c3_g1 | Genetic Information Processing | Folding, sorting and degradation | Protein processing in endoplasmic reticulum | ko04141 | K09487 | heat shock protein 90kDa beta |
| DN92570c1_g1 | Genetic Information Processing | Folding, sorting and degradation | Protein processing in endoplasmic reticulum | ko04141 | K09487 | heat shock protein 90kDa beta |
| DN59305c0_g1 | Genetic Information Processing | Folding, sorting and degradation | Protein processing in endoplasmic reticulum | ko04141 | K09503 | DnaJ homolog subfamily A member 2 |
| DN62275c0_g1 | Genetic Information Processing | Folding, sorting and degradation | Protein processing in endoplasmic reticulum | ko04141 | K09503 | DnaJ homolog subfamily A member 2 |
| DN57042c2_g3 | Genetic Information Processing | Folding, sorting and degradation | Protein processing in endoplasmic reticulum | ko04141 | K09503 | DnaJ homolog subfamily A member 2 |
| DN57042c2_g2 | Genetic Information Processing | Folding, sorting and degradation | Protein processing in endoplasmic reticulum | ko04141 | K09503 | DnaJ homolog subfamily A member 2 |
| DN60531c0_g1 | Genetic Information Processing | Folding, sorting and degradation | Protein processing in endoplasmic reticulum | ko04141 | K13525 | transitional endoplasmic reticulum ATPase |
| DN88420c1_g3 | Genetic Information Processing | Folding, sorting and degradation | Protein processing in endoplasmic reticulum | ko04141 | K13719 | ubiquitin thioesterase OTU1 |
| DN89334c0_g1 | Genetic Information Processing | Folding, sorting and degradation | Protein processing in endoplasmic reticulum | ko04141 | K13993 | HSP20 family protein |
| DN57227c2_g1 | Genetic Information Processing | Folding, sorting and degradation | Protein processing in endoplasmic reticulum | ko04141 | K13993 | HSP20 family protein |
| DN76447c0_g1 | Genetic Information Processing | Folding, sorting and degradation | Protein processing in endoplasmic reticulum | ko04141 | K13993 | HSP20 family protein |
| DN73502c0_g1 | Genetic Information Processing | Folding, sorting and degradation | Protein processing in endoplasmic reticulum | ko04141 | K13993 | HSP20 family protein |
| DN61517c2_g1 | Genetic Information Processing | Folding, sorting and degradation | Protein processing in endoplasmic reticulum | ko04141 | K13993 | HSP20 family protein |
| DN59213c0_g1 | Genetic Information Processing | Folding, sorting and degradation | Protein processing in endoplasmic reticulum | ko04141 | K13993 | HSP20 family protein |
| DN93729c4_g1 | Genetic Information Processing | Folding, sorting and degradation | Protein processing in endoplasmic reticulum | ko04141 | K13993 | HSP20 family protein |
| DN58615c1_g1 | Genetic Information Processing | Folding, sorting and degradation | Protein processing in endoplasmic reticulum | ko04141 | K13993 | HSP20 family protein |
| DN81535c3_g2 | Genetic Information Processing | Replication and repair | Nucleotide excision repair | ko03420 | K03868 | RING-box protein 1 |
| DN76620c0_g1 | Genetic Information Processing | Transcription | Spliceosome | ko03040 | K03283 | heat shock 70kDa protein 1/8 |
| DN77812c0_g1 | Genetic Information Processing | Transcription | Spliceosome | ko03040 | K03283 | heat shock 70kDa protein 1/8 |
| DN93137c2_g1 | Genetic Information Processing | Transcription | Spliceosome | ko03040 | K03283 | heat shock 70kDa protein 1/8 |
| DN58952c0_g5 | Genetic Information Processing | Transcription | Spliceosome | ko03040 | K03283 | heat shock 70kDa protein 1/8 |
| DN84857c0_g2 | Genetic Information Processing | Transcription | Spliceosome | ko03040 | K03283 | heat shock 70kDa protein 1/8 |
| DN56932c1_g2 | Genetic Information Processing | Transcription | Spliceosome | ko03040 | K03283 | heat shock 70kDa protein 1/8 |
| DN95595c1_g1 | Genetic Information Processing | Transcription | Spliceosome | ko03040 | K12891 | splicing factor, arginine/serine-rich 2 |
| DN90400c1_g1 | Genetic Information Processing | Transcription | Spliceosome | ko03040 | K12900 | FUS-interacting serine-arginine-rich protein 1 |
| DN89879c1_g1 | Metabolism | Amino acid metabolism | Alanine, aspartate and glutamate metabolism | ko00250 | K13566 | omega-amidase |
| DN93777c1_g3 | Metabolism | Amino acid metabolism | Cysteine and methionine metabolism | ko00270 | K00549 | 5-methyltetrahydropteroyltriglutamate--homocysteine methyltransferase |
| DN71986c0_g3 | Metabolism | Amino acid metabolism | Cysteine and methionine metabolism | ko00270 | K00549 | 5-methyltetrahydropteroyltriglutamate--homocysteine methyltransferase |
| DN92903c0_g1 | Metabolism | Amino acid metabolism | Cysteine and methionine metabolism | ko00270 | K00640 | serine O-acetyltransferase |
| DN72445c2_g1 | Metabolism | Amino acid metabolism | Cysteine and methionine metabolism | ko00270 | K00789 | S-adenosylmethionine synthetase |
| DN86067c1_g2 | Metabolism | Amino acid metabolism | Valine, leucine and isoleucine degradation | ko00280 | K00167 | 2-oxoisovalerate dehydrogenase E1 component beta subunit |
| DN93133c1_g3 | Metabolism | Amino acid metabolism | Valine, leucine and isoleucine degradation | ko00280 | K00626 | acetyl-CoA C-acetyltransferase |
| DN93133c1_g3 | Metabolism | Amino acid metabolism | Lysine degradation | ko00310 | K00626 | acetyl-CoA C-acetyltransferase |
| DN71981c1_g1 | Metabolism | Amino acid metabolism | Tyrosine metabolism | ko00350 | K01555 | fumarylacetoacetase |
| DN93133c1_g3 | Metabolism | Amino acid metabolism | Tryptophan metabolism | ko00380 | K00626 | acetyl-CoA C-acetyltransferase |
| DN80798c0_g1 | Metabolism | Amino acid metabolism | Tryptophan metabolism | ko00380 | K11820 | N-hydroxythioamide S-beta-glucosyltransferase |
| DN93802c0_g4 | Metabolism | Amino acid metabolism | Phenylalanine, tyrosine and tryptophan biosynthesis | ko00400 | K01657 | anthranilate synthase component I |
| DN80798c0_g1 | Metabolism | Biosynthesis of other secondary metabolites | Glucosinolate biosynthesis | ko00966 | K11820 | N-hydroxythioamide S-beta-glucosyltransferase |
| DN67844c1_g2 | Metabolism | Carbohydrate metabolism | Glycolysis / Gluconeogenesis | ko00010 | K01785 | aldose 1-epimerase |
| DN94353c2_g1 | Metabolism | Carbohydrate metabolism | Glycolysis / Gluconeogenesis | ko00010 | K01803 | triosephosphate isomerase (TIM) |
| DN68427c1_g1 | Metabolism | Carbohydrate metabolism | Pentose phosphate pathway | ko00030 | K01057 | 6-phosphogluconolactonase |
| DN78585c3_g1 | Metabolism | Carbohydrate metabolism | Pentose and glucuronate interconversions | ko00040 | K00012 | UDPglucose 6-dehydrogenase |
| DN94353c2_g1 | Metabolism | Carbohydrate metabolism | Fructose and mannose metabolism | ko00051 | K01803 | triosephosphate isomerase (TIM) |
| DN96180c7_g2 | Metabolism | Carbohydrate metabolism | Galactose metabolism | ko00052 | K01193 | beta-fructofuranosidase |
| DN67844c1_g2 | Metabolism | Carbohydrate metabolism | Galactose metabolism | ko00052 | K01785 | aldose 1-epimerase |
| DN78585c3_g1 | Metabolism | Carbohydrate metabolism | Ascorbate and aldarate metabolism | ko00053 | K00012 | UDPglucose 6-dehydrogenase |
| DN64366c1_g1 | Metabolism | Carbohydrate metabolism | Ascorbate and aldarate metabolism | ko00053 | K00434 | L-ascorbate peroxidase |
| DN59354c0_g1 | Metabolism | Carbohydrate metabolism | Ascorbate and aldarate metabolism | ko00053 | K00434 | L-ascorbate peroxidase |
| DN64366c2_g1 | Metabolism | Carbohydrate metabolism | Ascorbate and aldarate metabolism | ko00053 | K00434 | L-ascorbate peroxidase |
| DN66861c0_g1 | Metabolism | Carbohydrate metabolism | Ascorbate and aldarate metabolism | ko00053 | K14190 | GDP-L-galactose phosphorylase |
| DN78585c3_g1 | Metabolism | Carbohydrate metabolism | Starch and sucrose metabolism | ko00500 | K00012 | UDPglucose 6-dehydrogenase |
| DN96180c7_g2 | Metabolism | Carbohydrate metabolism | Starch and sucrose metabolism | ko00500 | K01193 | beta-fructofuranosidase |
| DN78585c3_g1 | Metabolism | Carbohydrate metabolism | Amino sugar and nucleotide sugar metabolism | ko00520 | K00012 | UDPglucose 6-dehydrogenase |
| DN76362c1_g7 | Metabolism | Carbohydrate metabolism | Amino sugar and nucleotide sugar metabolism | ko00520 | K13379 | reversibly glycosylated polypeptide / UDP-arabinopyranose mutase |
| DN94353c2_g1 | Metabolism | Carbohydrate metabolism | Inositol phosphate metabolism | ko00562 | K01803 | triosephosphate isomerase (TIM) |
| DN93133c1_g3 | Metabolism | Carbohydrate metabolism | Pyruvate metabolism | ko00620 | K00626 | acetyl-CoA C-acetyltransferase |
| DN93133c1_g3 | Metabolism | Carbohydrate metabolism | Glyoxylate and dicarboxylate metabolism | ko00630 | K00626 | acetyl-CoA C-acetyltransferase |
| DN93133c1_g3 | Metabolism | Carbohydrate metabolism | Propanoate metabolism | ko00640 | K00626 | acetyl-CoA C-acetyltransferase |
| DN93133c1_g3 | Metabolism | Carbohydrate metabolism | Butanoate metabolism | ko00650 | K00626 | acetyl-CoA C-acetyltransferase |
| DN84126c3_g3 | Metabolism | Energy metabolism | Oxidative phosphorylation | ko00190 | K02266 | cytochrome c oxidase subunit 6a |
| DN75937c0_g1 | Metabolism | Energy metabolism | Oxidative phosphorylation | ko00190 | K03943 | NADH dehydrogenase (ubiquinone) flavoprotein 2 |
| DN94353c2_g1 | Metabolism | Energy metabolism | Carbon fixation in photosynthetic organisms | ko00710 | K01803 | triosephosphate isomerase (TIM) |
| DN92903c0_g1 | Metabolism | Energy metabolism | Sulfur metabolism | ko00920 | K00640 | serine O-acetyltransferase |
| DN93133c1_g3 | Metabolism | Lipid metabolism | Fatty acid degradation | ko00071 | K00626 | acetyl-CoA C-acetyltransferase |
| DN93133c1_g3 | Metabolism | Lipid metabolism | Synthesis and degradation of ketone bodies | ko00072 | K00626 | acetyl-CoA C-acetyltransferase |
| DN70026c3_g1 | Metabolism | Lipid metabolism | Steroid biosynthesis | ko00100 | K00511 | squalene monooxygenase |
| DN89411c2_g1 | Metabolism | Lipid metabolism | Steroid biosynthesis | ko00100 | K00511 | squalene monooxygenase |
| DN74602c0_g1 | Metabolism | Metabolism of cofactors and vitamins | Pantothenate and CoA biosynthesis | ko00770 | K09680 | type II pantothenate kinase |
| DN93777c1_g3 | Metabolism | Metabolism of other amino acids | Selenocompound metabolism | ko00450 | K00549 | 5-methyltetrahydropteroyltriglutamate--homocysteine methyltransferase |
| DN71986c0_g3 | Metabolism | Metabolism of other amino acids | Selenocompound metabolism | ko00450 | K00549 | 5-methyltetrahydropteroyltriglutamate--homocysteine methyltransferase |
| DN76507c0_g2 | Metabolism | Metabolism of other amino acids | Glutathione metabolism | ko00480 | K00383 | glutathione reductase (NADPH) |
| DN64366c1_g1 | Metabolism | Metabolism of other amino acids | Glutathione metabolism | ko00480 | K00434 | L-ascorbate peroxidase |
| DN59354c0_g1 | Metabolism | Metabolism of other amino acids | Glutathione metabolism | ko00480 | K00434 | L-ascorbate peroxidase |
| DN64366c2_g1 | Metabolism | Metabolism of other amino acids | Glutathione metabolism | ko00480 | K00434 | L-ascorbate peroxidase |
| DN93133c1_g3 | Metabolism | Metabolism of terpenoids and polyketides | Terpenoid backbone biosynthesis | ko00900 | K00626 | acetyl-CoA C-acetyltransferase |
| DN92746c2_g1 | Metabolism | Metabolism of terpenoids and polyketides | Terpenoid backbone biosynthesis | ko00900 | K03526 | (E)-4-hydroxy-3-methylbut-2-enyl-diphosphate synthase |
| DN62254c4_g1 | Metabolism | Metabolism of terpenoids and polyketides | Terpenoid backbone biosynthesis | ko00900 | K03526 | (E)-4-hydroxy-3-methylbut-2-enyl-diphosphate synthase |
| DN70026c3_g1 | Metabolism | Metabolism of terpenoids and polyketides | Sesquiterpenoid and triterpenoid biosynthesis | ko00909 | K00511 | squalene monooxygenase |
| DN89411c2_g1 | Metabolism | Metabolism of terpenoids and polyketides | Sesquiterpenoid and triterpenoid biosynthesis | ko00909 | K00511 | squalene monooxygenase |
| DN93133c1_g3 | Metabolism | Overview | Carbon metabolism | ko01200 | K00626 | acetyl-CoA C-acetyltransferase |
| DN92903c0_g1 | Metabolism | Overview | Carbon metabolism | ko01200 | K00640 | serine O-acetyltransferase |
| DN68427c1_g1 | Metabolism | Overview | Carbon metabolism | ko01200 | K01057 | 6-phosphogluconolactonase |
| DN94353c2_g1 | Metabolism | Overview | Carbon metabolism | ko01200 | K01803 | triosephosphate isomerase (TIM) |
| DN80798c0_g1 | Metabolism | Overview | 2-Oxocarboxylic acid metabolism | ko01210 | K11820 | N-hydroxythioamide S-beta-glucosyltransferase |
| DN93133c1_g3 | Metabolism | Overview | Fatty acid metabolism | ko01212 | K00626 | acetyl-CoA C-acetyltransferase |
| DN93777c1_g3 | Metabolism | Overview | Biosynthesis of amino acids | ko01230 | K00549 | 5-methyltetrahydropteroyltriglutamate--homocysteine methyltransferase |
| DN71986c0_g3 | Metabolism | Overview | Biosynthesis of amino acids | ko01230 | K00549 | 5-methyltetrahydropteroyltriglutamate--homocysteine methyltransferase |
| DN92903c0_g1 | Metabolism | Overview | Biosynthesis of amino acids | ko01230 | K00640 | serine O-acetyltransferase |
| DN72445c2_g1 | Metabolism | Overview | Biosynthesis of amino acids | ko01230 | K00789 | S-adenosylmethionine synthetase |
| DN93802c0_g4 | Metabolism | Overview | Biosynthesis of amino acids | ko01230 | K01657 | anthranilate synthase component I |
| DN94353c2_g1 | Metabolism | Overview | Biosynthesis of amino acids | ko01230 | K01803 | triosephosphate isomerase (TIM) |
| DN84227c0_g3 | Organismal Systems | Environmental adaptation | Plant-pathogen interaction | ko04626 | K09487 | heat shock protein 90kDa beta |
| DN76833c3_g1 | Organismal Systems | Environmental adaptation | Plant-pathogen interaction | ko04626 | K09487 | heat shock protein 90kDa beta |
| DN92570c1_g1 | Organismal Systems | Environmental adaptation | Plant-pathogen interaction | ko04626 | K09487 | heat shock protein 90kDa beta |
| DN95581c2_g1 | Organismal Systems | Environmental adaptation | Plant-pathogen interaction | ko04626 | K13412 | calcium-dependent protein kinase |
| DN77225c1_g1 | Organismal Systems | Environmental adaptation | Plant-pathogen interaction | ko04626 | K13412 | calcium-dependent protein kinase |
| DN61976c0_g1 | Organismal Systems | Environmental adaptation | Plant-pathogen interaction | ko04626 | K13448 | calcium-binding protein CML |
| DN77244c0_g2 | Organismal Systems | Environmental adaptation | Plant-pathogen interaction | ko04626 | K09422 | myb proto-oncogene protein, plant |
| DN72991c0_g1 | Organismal Systems | Environmental adaptation | Plant-pathogen interaction | ko04626 | K09422 | myb proto-oncogene protein, plant |
| DN62285c1_g3 | Organismal Systems | Environmental adaptation | Plant-pathogen interaction | ko04626 | K09422 | myb proto-oncogene protein, plant |
| DN84109c0_g2 | Organismal Systems | Environmental adaptation | Circadian rhythm - plant | ko04712 | K12118 | cryptochrome 1 |
| DN83841c0_g1 | Organismal Systems | Environmental adaptation | Circadian rhythm - plant | ko04712 | K12125 | protein EARLY FLOWERING 3 |
